# Dynamic Changes and Future Trend Forecasts in the Global Burden of Guillain–Barré Syndrome: Analysis of 204 Countries and Regions From 1990 to 2021, Including the Impact of the COVID‐19 Pandemic

**DOI:** 10.1002/iid3.70473

**Published:** 2026-06-17

**Authors:** Qingyu Song, Mengmeng Wang, Zheng Shen, Zhaoyi Jing, Yibin Fan, Xiaojin Zhang, Li Huang, Wen Li, Yin Shi, Dianhui Yang

**Affiliations:** ^1^ The Seventh Clinical College Shanghai University of Traditional Chinese Medicine Shanghai China; ^2^ College of Acupuncture and Massage Shandong University of Traditional Chinese Medicine Jinan China; ^3^ Acupuncture Department Affiliated Hospital of Shandong University of Traditional Chinese Medicine Jinan China; ^4^ The First Clinical College Shandong University of Traditional Chinese Medicine Jinan China; ^5^ Acupuncture Department Seventh People's Hospital Affiliated to Shanghai University of Traditional Chinese Medicine Jinan China

**Keywords:** COVID‐19, decomposition analysis, global burden of disease, Guillain–Barré syndrome, inequality analysis

## Abstract

**Background:**

Guillain–Barré syndrome (GBS) is an immune‐mediated neuropathy that may be influenced by infectious, demographic, and healthcare‐related factors. Although increasing attention has been paid to neurological complications during the COVID‐19 pandemic, the global burden, inequalities, and future trends of GBS remain insufficiently characterized.

**Methods:**

This population‐based observational epidemiological study used data from the Global Burden of Disease Study 2021 to assess the burden of GBS across 204 countries and territories from 1990 to 2021. The main outcomes included prevalence, years lived with disability (YLDs), age‐standardized prevalence rate (ASPR), age‐standardized YLD rate (ASYR), and estimated annual percentage change (EAPC). Analyses were stratified by sex, age group, country, region, and socio‐demographic index (SDI). Inequality analysis, decomposition analysis, Joinpoint regression, and Bayesian age‐period‐cohort modeling were further used to assess socioeconomic disparities, driving factors, temporal changes, and future trends.

**Results:**

From 1990 to 2021, the global burden of GBS increased substantially. In 2021, the global number of prevalent cases reached 471,850, and the ASPR was 5.91 per 100,000 population. The corresponding number of YLDs was 139,639, with an ASYR of 1.75 per 100,000 population. The burden was lowest among children younger than 5 years and increased progressively with age. Males generally had a higher burden than females, particularly among older adults. Low‐SDI regions experienced disproportionately higher ASPR and ASYR, indicating substantial global inequalities. The increases observed after 2019 temporally coincided with the COVID‐19 pandemic; however, this association should be interpreted cautiously. Decomposition analysis suggested that epidemiological changes, population growth, and population aging collectively contributed to the increasing burden of GBS.

**Conclusion:**

The global burden of GBS increased markedly from 1990 to 2021, with substantial regional, sex‐, age‐, and SDI‐related heterogeneity. The post‐2019 increase temporally coincided with the COVID‐19 pandemic, but causal inference should be avoided due to the observational nature of this study. Strengthening neurological surveillance, improving early diagnosis and treatment capacity, and reducing healthcare disparities in low‐SDI regions may help mitigate the future burden of GBS.

**Abbreviations**
ASPR: age‐standardized prevalence rate
ASR: age‐standardized rate
ASYR: age‐standardized years lived with disability rate
BAPC: Bayesian age‐period‐cohort
CI: confidence interval
EAPC: estimated annual percentage changes
GBD: Global Burden of Disease
GBS: Guillain–Barré syndrome
SARS‐CoV‐2: severe acute respiratory syndrome coronavirus 2
SDI: socio‐demographic Index
SII: inequality slope index
UI: uncertainty interval
WHO: World Health Organization
YLD: years lived with disability

## 1 Introduction

Guillain–Barré syndrome (GBS) is an immune‐mediated polyneuropathy caused by infection or other triggering factors. Its main clinical manifestations include symmetrical flaccid muscle weakness in the limbs, often accompanied by sensory abnormalities and weakened or absent tendon reflexes [1, 2]. Approximately 20% of GBS patients face death or severe disability [3, 4]. Among them, elderly patients and those with axonal involvement have a more severe prognosis [5]. GBS has a widespread impact on regions and populations around the world. In‐depth research into differences in the disease burden of GBS in countries with varying levels of economic development is important for improving the efficiency and equity of global healthcare systems.

Severe acute respiratory syndrome coronavirus 2 (SARS‐CoV‐2) is the pathogen that causes COVID‐19. Research indicates that SARS‐CoV‐2 infection may increase the risk of developing GBS [6, 7, 8]. Compared to other health issues (such as cancer and cardiovascular disease), GBS is not a priority concern in the field of global public health [9]. The prevalence of GBS and years lived with disability (YLDs) rates are not only influenced by socio‐demographic index (SDI) levels, but are also closely related to public health policies and vaccination coverage in each country [10, 11]. In addition, economic progress and social changes have triggered major shifts in global population structure [12, 13], but the impact of these changes on the burden of GBS has not yet been addressed. Given the significant impact of GBS on individual health, social and public health, conducting in‐depth research on the epidemiological characteristics of GBS is conducive to monitoring the progress of disease burden on health outcomes.

## 2 Methods

### 2.1 Study Design and Data Source

This study was a population‐based observational epidemiological analysis based on the Global Burden of Disease (GBD) 2021 database. Data on GBS were obtained from the GBD 2021 study, coordinated by the Institute for Health Metrics and Evaluation, which systematically estimates disease burden across 204 countries and territories from 1990 to 2021.

GBS cases were identified using the International Classification of Diseases, Tenth Revision (ICD‐10) code G61.0. The GBD framework integrates multiple standardized data sources, including hospital records, surveillance systems, health insurance databases, published epidemiological studies, and vital registration systems. To improve comparability and reliability across countries and time periods, GBD 2021 applied advanced Bayesian statistical models, including DisMod‐MR 2.1, with standardized quality‐control procedures and uncertainty propagation methods. Detailed methodological information has been published previously.

### 2.2 Study Variables and Sociodemographic Classification

The primary outcome variables included prevalence, YLDs, age‐standardized prevalence rate (ASPR), age‐standardized YLD rate (ASYR), and estimated annual percentage change (EAPC). All age‐standardized rates (ASRs) were expressed per 100,000 population.

Analyses were stratified by sex, age group, country, region, and SDI level. The SDI is a composite indicator developed within the GBD framework based on income per capita, educational attainment, and fertility rate among individuals younger than 25 years. Countries and territories were categorized into five SDI regions: low, low‐middle, middle, high‐middle, and high SDI.

### 2.3 Temporal Trend Analysis

Temporal trends in GBS burden were assessed using EAPC, which was calculated from a log‐linear regression model:

ln(ASR)=α+βX+ε,

where ASR represents the ASR and *β* indicates the annual rate of change. EAPC was calculated as:

EAPC=100×(exp(β)−1).

An EAPC > 0 indicated an increasing trend, whereas an EAPC < 0 indicated a decreasing trend. Trends were considered statistically significant when the 95% confidence interval (CI) of the EAPC did not include zero.

Joinpoint regression analysis was additionally performed to identify significant temporal changes in GBS burden trends. Annual percentage change (APC) and average annual percentage change (AAPC) were estimated using permutation tests.

### 2.4 Correlation and Inequality Analyses

Spearman's rank correlation analysis was conducted to evaluate associations between SDI and ASRs at both regional and national levels. Correlation coefficients (*R*) and corresponding *p*‐values were reported.

Health inequalities associated with GBS burden were assessed using the slope index of inequality (SII) and concentration index (CI). Negative SII and CI values indicated a disproportionate concentration of disease burden among populations with lower SDI levels.

### 2.5 Decomposition Analysis

Decomposition analysis based on the Das Gupta framework was performed to quantify the relative contributions of population growth, population aging, and epidemiological changes to changes in GBS prevalence and YLDs between 1990 and 2021. This method decomposes the net change in disease burden into independent demographic and epidemiological components, thereby allowing assessment of the primary drivers underlying temporal changes.

### 2.6 Forecasting Analysis

Future trends in GBS burden from 2022 to 2035 were projected using Bayesian age‐period‐cohort (BAPC) models implemented with integrated nested Laplace approximations (INLA). The BAPC framework incorporates age, period, and cohort effects simultaneously and has been widely used in disease burden forecasting studies. Forecast estimates were presented with corresponding 95% uncertainty intervals (UIs).

### 2.7 Statistical Software and Significance

All statistical analyses and visualizations were performed using R software (version 4.5.1) and JD_GBDR (v2.7.6; Jingding Medical Technology Co. Ltd.). Major packages included ggplot2 for visualization, segmented and Joinpoint for trend analysis, and BAPC and INLA for Bayesian forecasting analyses. All statistical tests were two‐sided, and *p* < 0.05 was considered statistically significant.

### 2.8 Ethical Statement

This study used publicly available de‐identified data from the GBD 2021 database; therefore, ethical approval and informed consent were not required.

## 3 Results

### 3.1 The Burden of Disease of GBS

#### 3.1.1 Global Trends

The number of global GBS cases increased significantly from 96,819 cases in 1990 (95% UI: 77,059–121,121) to 471,850 cases in 2021 (95% UI: 389,187–554,145) (Supporting Information S5: Table S1). while the ASPR increased from 1.93 (95% UI: 1.54–2.38) per 100,000 population in 1990 to 5.91 per 100,000 population (95% UI: 4.87–6.97) in 2021, with an EAPC of 0.95 (95% CI: 0.53–1.37). In 2021, the number of YLDs for GBS was 139,639 cases (95% UI: 90,387–202,387), the ASYR was 1.75 cases per 100,000 people (95% CI: 1.12–2.54), and the EAPC was [0.95 (95% CI: 0.53–1.36)].

#### 3.1.2 SDI Regional Level

**Figure 1:**
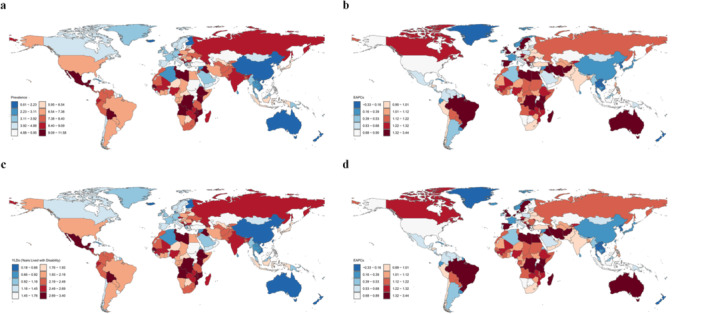
Global distribution of Guillain–Barré syndrome burden in 2021. (a and b) Age‐standardized prevalence rate; (c and d) Age‐standardized YLDs rate.

In 2021, low‐SDI regions bore a disproportionately high burden of GBS, with 82,360 prevalent cases (95% UI: 65,096–99,251) and 24,376 YLDs (95% UI: 15,135–35,236). These regions also exhibited the highest ASPR and ASYR, reaching 8.03 per 100,000 population (95% UI: 6.39–9.60) and 2.37 per 100,000 population (95% UI: 1.49–3.44), respectively (Supporting Information S5: Table S1, Figure 1, and Supporting Information S1: Figure S1). In contrast, the high‐middle SDI region showed the lowest ASPR and ASYR, at 3.82 per 100,000 population (95% UI: 3.16–4.56) and 1.13 per 100,000 population (95% UI: 0.72–1.63), respectively. From 1990 to 2021, the middle SDI region experienced the most pronounced increases in both ASPR and ASYR, with EAPCs of 1.18 (95% CI: 0.78–1.59) for both indicators. Conversely, the high SDI region demonstrated the smallest increases, with corresponding EAPCs of 0.45 (95% CI: 0.22–0.67).

#### 3.1.3 21 Geographical Areas Changed

Among the 21 GBD regions, South Asia bore the highest absolute burden of GBS in 2021, with 155,150 prevalent cases (95% UI: 126,682–185,328) and 45,945 YLDs (95% UI: 28,849–68,526) (Supporting Information S5: Table S1 and Figure 1). In contrast, Central Latin America exhibited the highest age‐standardized burden, with an ASPR of 9.49 per 100,000 population (95% UI: 7.97–11.13) and an ASYR of 2.81 per 100,000 population (95% UI: 1.78–4.05). Conversely, East Asia showed the lowest ASPR and ASYR, at 0.63 per 100,000 population (95% UI: 0.48–0.82) and 0.19 per 100,000 population (95% UI: 0.12–0.29), respectively. From 1990 to 2021, all regions demonstrated increasing trends in GBS burden, although the magnitude of increase varied substantially across regions. Tropical Latin America showed the most pronounced increases in both ASPR and ASYR, with corresponding EAPCs of 1.34 (95% CI: 0.66–2.02), whereas High‐income Asia Pacific exhibited the smallest increases, with EAPCs of 0.17 (95% CI: −0.00 to 0.34) for ASPR and 0.17 (95% CI: −0.01 to 0.34) for ASYR.

#### 3.1.4 National Level

At the national level, India bore the highest absolute burden of GBS in 2021, with 120,439 prevalent cases (95% UI: 98,026–144,220) and 35,666 YLDs (95% UI: 22,496–52,522) (Supporting Information S5: Table S1 and Figure 1). In contrast, North Macedonia exhibited the highest age‐standardized burden worldwide, with an ASPR of 11.47 per 100,000 population (95% UI: 8.21–14.48) and an ASYR of 3.37 per 100,000 population (95% UI: 1.99–5.11). From 1990 to 2021, most countries showed increasing trends in GBS burden. Only in New Zealand and Puerto Rico did the ASPR and ASYR show a downward trend. Specifically, the EAPC in New Zealand was [−0.16 (95% CI: −0.36 to 0.04)] and [−0.16 (95% CI: −0.36 to 0.05)], while the EAPC in Puerto Rico was [−0.33 (95% CI: −0.72 to 0.06)] and [−0.33 (95% CI: −0.73 to 0.06)]. Conversely, the United Kingdom exhibited the most pronounced increases in both ASPR and ASYR, with corresponding EAPCs of 3.41 (95% CI: 2.93–3.89) and 3.41 (95% CI: 2.94–3.89), respectively.

### 3.2 Age and Gender Patterns and Overall Time Trends

In 2021, the burden of GBS disease among children under 5 years old was the lowest, and the burden level increased with age (Supporting Information S2: Figure S2). The age‐specific pattern of YLDs worldwide was consistent with the trend in prevalence. The overall disease burden for men is significantly higher than that for women, and this difference is particularly pronounced among the elderly population.

### 3.3 Correlation Between SDI and Disease Burden

**Figure 2:**
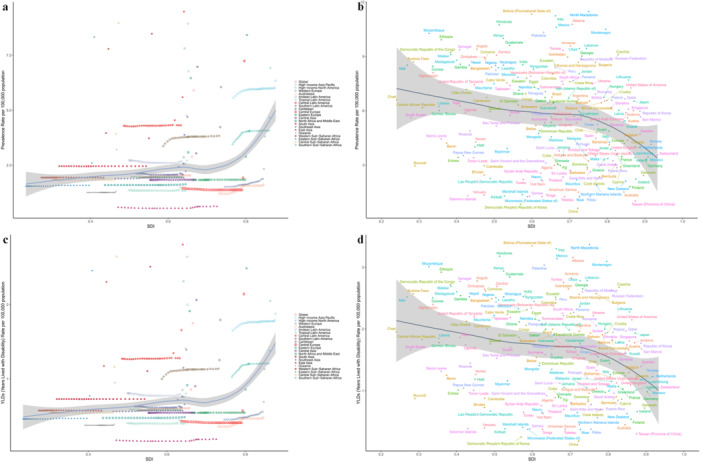
Correlation between ASR of Guillain–Barré syndrome and SDI at the national and regional levels in 2021. (a and b) Age‐standardized prevalence rate; (c and d) Age‐standardized YLDs rate.

The research found that SDI was positively correlated with both ASPR and ASYR at the regional level, with correlation coefficients of *R* = 0.1828 (*p* < 0.001) and *R* = 0.1829 (*p* < 0.001), respectively (Figure 2). Conversely, when analyzed at the country level, SDI was found to be negatively correlated with ASPR (*R* = −0.2700, *p* < 0.001) and ASYR (*R* = −0.2713, *p* < 0.001).

### 3.4 Inequality Analysis

**Figure 3:**
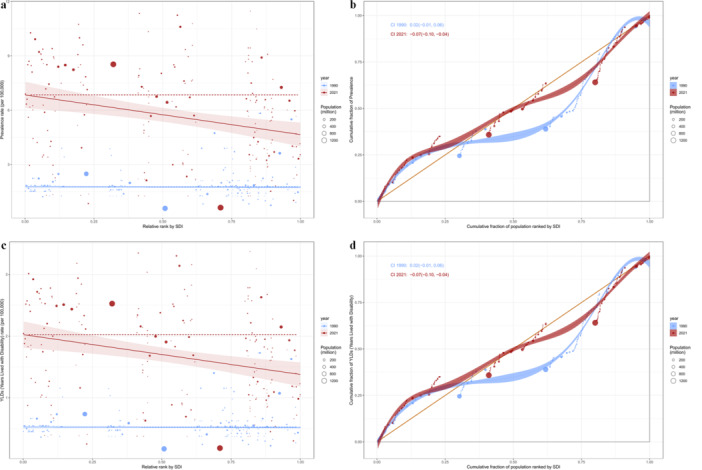
SDI‐related health inequality regression curves and concentration curves for the global burden of Guillain–Barré syndrome, 1990 and 2021. The health inequality regression curve on the left and the concentration curve on the right; (a and b) Age‐standardized prevalence rate; (c and d) Age‐standardized YLDs rate.

As measured by the slope index of inequality, the ASPR was observed to shift from −0.03 (95% CI: −0.20 to 0.13) in 1990 to −2.19 (95% CI: −3.34 to −1.04) in 2021 (Figure 3). A similar pattern was indicated by the relative concentration index, through which ASPR was shown to change from 0.02 (95% CI: −0.01 to 0.06) in 1990 to −0.07 (95% CI: −0.10 to −0.04) in 2021. A consistent trend over the same period was also exhibited by the ASYR.

### 3.5 Join‐Point Regression Analysis

**Figure 4:**
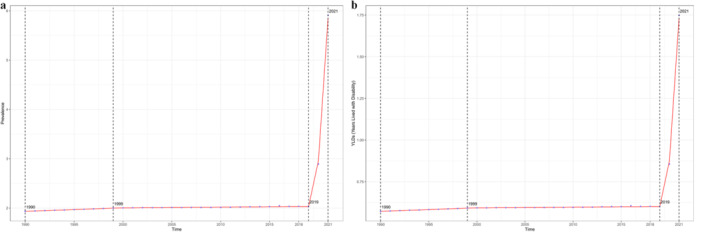
Join‐point regression analysis of temporal trends in the burden of Guillain–Barré syndrome from 1990 to 2021. (a) Age‐standardized prevalence rate; (b) Age‐standardized YLDs rate.

Both the ASPR and ASYR for GBS worldwide showed an upward trend, with AAPC values of [5.06 (95% CI: 4.91–5.22; *p* < 0.05)] and [5.06 (95% CI: 4.91–5.21; *p* < 0.05)], respectively (Figure 4). The most significant increases in ASPR and ASYR occurred between 2019 and 2021, with APC values of [98.04 (95% CI: 95.12–101.00; *p* < 0.05)] and [97.97 (95% CI: 95.07–100.92; *p* < 0.001)], respectively. It is worth noting that the extremely high disability rate that emerged during the COVID‐19 pandemic is likely related to the complications caused by severe infection with the novel coronavirus, rather than being solely associated with GBS.

### 3.6 Decomposition Analysis

The increase in the number of global GBS cases was the result of the combined effects of epidemiological changes (70.36%), population growth (26.56%), and aging (3.08%) (Supporting Information S3: Figure S3). The increase in global YLDs is similarly driven by epidemiological changes (70.36%), population growth (26.57%), and aging (3.07%); additionally, the patterns of case numbers and YLDs growth in the five SDI regions align with the global trends.

### 3.7 Predicted Trends in the Burden of GBS Disease

By 2035, the global ASPR and ASYR for GBS are projected to reach 8.25 per 100,000 population (95% UI: −0.80 to 17.30) and 2.48 per 100,000 population (95% UI: −0.28 to 5.24), respectively, indicating that the disease burden of GBS will continue to rise (Supporting Information S4: Figure S4).

## 4 Discussion

### 4.1 Potential Impact of the COVID‐19 Pandemic on GBS Burden

The relationship between GBS and SARS‐CoV‐2 infection remains controversial. Although several observational studies have suggested a possible association between COVID‐19 and increased GBS incidence or severity [7, 8, 14], other epidemiological investigations have reported little or no significant increase in GBS occurrence during the pandemic period [15, 16]. Therefore, the increase in the global burden of GBS observed between 2019 and 2021 in the present study should be interpreted cautiously. Our findings demonstrated marked increases in both ASPR and ASYR after 2019, temporally coinciding with the COVID‐19 pandemic; however, this temporal association does not necessarily establish a direct causal relationship between SARS‐CoV‐2 infection and GBS. GBS is a complex immune‐mediated neuropathy with multifactorial pathogenesis involving molecular mimicry, dysregulated immune activation, genetic susceptibility, and post‐infectious immune responses [1, 2]. Consequently, changes in communicable disease exposure alone are unlikely to fully explain the substantial increase in GBS burden observed in recent years. Several mechanisms may have contributed to the apparent increase in GBS burden during the pandemic period. SARS‐CoV‐2 infection has been proposed as a potential trigger for immune‐mediated neurological injury through inflammatory and autoimmune pathways [14, 17], while COVID‐19‐related GBS cases may present with more severe manifestations, including respiratory dysfunction and systemic complications, potentially leading to higher disability burdens and healthcare utilization [7, 18]. In addition, pandemic‐related healthcare disruptions, altered healthcare‐seeking behavior, changes in referral patterns, and increased neurological surveillance may also have influenced GBS diagnosis and reporting. Importantly, the increase in reported GBS burden after 2019 may partially reflect improved recognition and diagnosis of mild or atypical neurological manifestations during the pandemic rather than a true biological increase in disease occurrence. Increased awareness of neurological complications associated with COVID‐19 may therefore have contributed to enhanced detection and reporting of GBS cases worldwide. Further large‐scale prospective and mechanistic studies are needed to clarify the long‐term relationship between COVID‐19 and GBS, while strengthening neurological surveillance systems and improving early diagnosis and treatment capacity may help reduce the future burden of GBS.

### 4.2 Global Inequality of GBS

Our findings revealed substantial global inequalities in the burden of GBS, with low‐SDI regions experiencing disproportionately higher ASPR and ASYR. Multiple structural and healthcare‐related factors may contribute to these disparities. In low‐SDI settings, poor sanitation conditions, higher exposure to infectious diseases, limited healthcare accessibility, delayed diagnosis, and insufficient treatment resources may collectively increase the disability burden associated with GBS [19, 20, 21, 22]. However, given the complex immune‐mediated pathogenesis of GBS, these socioeconomic and environmental factors alone are unlikely to fully explain regional differences in disease burden. Variations in healthcare infrastructure, neurological surveillance capacity, diagnostic practices, and reporting systems may also contribute to the observed inequalities. The COVID‐19 pandemic may have further amplified these disparities, as many low‐SDI countries experienced shortages of healthcare personnel, limited intensive care capacity, unequal vaccine distribution, and disruptions to routine neurological services during the pandemic period [23, 24, 25]. These systemic pressures may have affected both the diagnosis and management of GBS, potentially contributing to worsening health inequalities. At the national level, India exhibited the highest absolute burden of GBS in terms of both prevalent cases and YLDs, which likely reflects the combined effects of large population size, high population density, regional disparities in sanitation conditions, and unequal access to healthcare services [26, 27, 28, 29, 30]. In contrast, North Macedonia showed the highest ASPR and ASYR worldwide. Although the underlying reasons for this elevated age‐standardized burden remain unclear, differences in population structure, diagnostic practices, healthcare utilization, or surveillance systems may partially contribute to these findings and warrant further investigation. Interestingly, only New Zealand and Puerto Rico demonstrated decreasing trends in ASPR and ASYR during the study period. While the mechanisms underlying these favorable trends remain uncertain, relatively accessible healthcare systems, effective infectious disease control, and stronger public health infrastructure may have contributed to these observations [31, 32]. Future studies incorporating country‐specific epidemiological and healthcare system data are needed to better understand the mechanisms underlying regional and national heterogeneity in GBS burden.

### 4.3 The Age and Gender Distribution Characteristics of the Burden of GBS Disease

The age‐ and sex‐specific patterns observed in this study were generally consistent with previous epidemiological studies of GBS [3]. The burden of GBS was lowest among children younger than 5 years and increased progressively with age, while males consistently exhibited a higher burden than females across most age groups, particularly among older populations. Several factors may contribute to these patterns. GBS is a complex immune‐mediated neuropathy that is often preceded by infectious or immune‐related triggers. The relatively lower burden observed in young children may be associated with differences in immune response patterns, lower cumulative exposure to environmental and infectious triggers, and potential protective effects during early childhood [33, 34]. In contrast, aging‐related immune dysregulation, increased comorbidity burden, and cumulative exposure to infectious agents may contribute to the higher burden observed in older adults [35, 36]. The higher burden among males may reflect multifactorial interactions involving immune regulation, hormonal influences, genetic susceptibility, and differences in environmental exposure [37]. However, the precise biological and epidemiological mechanisms underlying age‐ and sex‐related differences in GBS remain incompletely understood and warrant further investigation [38].

### 4.4 The Driving Factors of GBS Disease Burden and Predictions of Future Trends

Decomposition analysis indicated that epidemiological changes, population growth, and population aging contributed to the increasing global burden of GBS, with epidemiological changes accounting for the largest proportion of the observed increase. However, the factors underlying these epidemiological changes are likely multifactorial and may include changes in infectious disease exposure, improvements in diagnostic capacity, increased neurological surveillance, healthcare system changes during the COVID‐19 pandemic, and evolving demographic structures. Population growth and aging may further contribute to increasing absolute numbers of GBS cases and YLDs worldwide, particularly in regions undergoing rapid demographic transition.

Our projections suggest that the global burden of GBS may continue to increase through 2035, although the rate of increase is expected to gradually slow over time. Nevertheless, these predictions should be interpreted cautiously because future trends may be influenced by multiple uncertain factors, including changes in healthcare accessibility, diagnostic practices, infectious disease patterns, vaccination programs, and public health policies [37, 39]. In addition, improvements in neurological care, rehabilitation services, and healthcare infrastructure may alter the long‐term trajectory of GBS burden in different regions. Continued epidemiological surveillance and prospective studies will therefore be important for refining future burden estimates and understanding the evolving global patterns of GBS.

### 4.5 Translating Evidence Into Stratified Policy Action

To alleviate the burden of GBS and improve health equity, the following measures should be taken: (1) Strengthen the early identification and diagnosis standards of GBS, and establish a flexible medical resource allocation mechanism to cope with public health emergencies; (2) Increase medical investment and personnel training in low SDI countries, improve basic health conditions, and narrow the global treatment gap; (3) Conduct in‐depth research on the pathogenesis of GBS, gender differences, and the causes in specific high‐burden regions, to provide evidence‐based support.

### 4.6 Strengths and Limitations

This study systematically evaluated temporal trends and potential epidemiological changes in the global burden of GBS during the COVID‐19 pandemic period, but also deeply analyzed multiple dimensions such as age, gender, and SDI. These findings provide important evidence for understanding global patterns and public health implications of GBS burden.

Several limitations should be considered. First, validation of GBD estimates depends on diverse data sources that vary in quality and completeness. Although the GBD modeling framework incorporates multiple validation steps—including internal consistency checks, comparison with alternative data sources, and expert clinical review—the estimates remain model‐based and subject to the quality of underlying inputs. Hospital‐based data sources, which constitute a substantial component of GBD inputs, may underrepresent the true population burden when healthcare access is constrained by financial, geographic, or cultural barriers, while referral bias may lead to overrepresentation of severe cases in some settings. Despite these limitations, the GBD methodology represents the most comprehensive and systematic synthesis of available global data, enabling standardized cross‐national and temporal comparisons. Future research incorporating primary epidemiological studies, particularly population‐based surveys in underrepresented regions, will be essential to validate and refine these estimates. In addition, the ecological and observational nature of this study does not permit causal inference regarding the relationship between COVID‐19 and GBS, and the observed temporal associations should therefore be interpreted cautiously. Second, predictions based on historical trends cannot fully reflect the future advancements in diagnostic technologies, therapeutic innovations, or changes in environmental exposures, which may influence the long‐term trajectory and uncertainty of future burden estimates. Although our research results provide important insights into the epidemiology of global GBS, the findings should be interpreted in the context of these methodological limitations and potential sources of bias.

## 5 Conclusion

The global burden of GBS increased substantially between 1990 and 2021, with notable regional, age‐, sex‐, and SDI‐related heterogeneity. The increases observed after 2019 temporally coincided with the COVID‐19 pandemic, although the relationship between COVID‐19 and GBS should be interpreted cautiously due to the observational nature of this study. Low‐SDI regions experienced disproportionately higher disease burden and health inequalities, highlighting the importance of improving healthcare accessibility, neurological surveillance, and early diagnosis and treatment capacity. Continued epidemiological research and strengthened public health strategies may help reduce the future global burden of GBS.

## Author Contributions

**Qingyu Song:** conceptualization, methodology, writing – original draft. **Mengmeng Wang:** writing – review and editing, data curation. **Zheng Shen:** formal analysis, visualization. **Zhaoyi Jing:** formal analysis, visualization. **Yibin Fan:** formal analysis, visualization. **Xiaojin Zhang:** conceptualization, methodology. **Li Huang:** conceptualization, methodology. **Wen Li:** methodology, conceptualization. **Yin Shi:** project administration, resources, supervision. **Dianhui Yang:** resources, project administration, writing – review and editing, supervision, funding acquisition.

## Disclosure

During the writing and revision process of this study, our team employed AI‐assisted tasks such as language polishing, logical organization, and expression optimization. However, the overall research concept, data analysis, and conclusions of the study were independently completed by our team. The AI did not participate in the core academic creation and decision‐making process. I am fully responsible for the authenticity, originality, and academic compliance of the paper content.

## Ethics Statement

The authors have nothing to report.

## Consent

The authors have nothing to report.

## Conflicts of Interest

The authors declare no conflicts of interest.

## Supporting information


**Figure S1:** Correlation between ASR of Guillain‐Barré syndrome and SDI at the national and regional levels in 2021. (a, b) Age‐standardized prevalence rate; (c, d) Age‐standardized YLDs rate.

**Figure S2:** Age distribution and trend of the Guillain‐Barré syndrome burden in 2021. (a) prevalent cases; (b) YLDs.

**Figure S3:** Key drivers of Guillain‐Barré syndrome burden at global and SDI levels from 1990 to 2021: population growth, ageing, and epidemiological changes. The black dots represent the sum of contributions to changes in all three factors. (a) prevalent cases; (b) YLDs.

**Figure S4:** Trends in the burden of Guillain‐Barré syndrome: observed rates (1990–2021) and predicted rates (2022–2035). The blue region shows the upper and lower limits of the 95% UI. (a) prevalent cases; (b) YLDs.

Supporting File

## Data Availability

The relevant data can be accessed using the Global Health Data Exchange tool (https://ghdx.healthdata.org/gbd-2021/sources). The prevalence and YLDs measurements for Guillain–Barré Syndrome at global, regional, and national levels from 1990 to 2021 were retrieved using the GBD Results Tool (https://vizhub.healthdata.org/gbd-results/).
