# Supplementary figures and images for: Dynamic Changes and Future Trend Forecasts in the Global Burden of Guillain–Barré Syndrome: Analysis of 204 Countries and Regions From 1990 to 2021, Including the Impact of the COVID‐19 Pandemic
Source: Immun Inflamm Dis. 2026 Jun 17;14(6):e70473. doi: 10.1002/iid3.70473 (PMC13276001; doi:10.1002/iid3.70473)

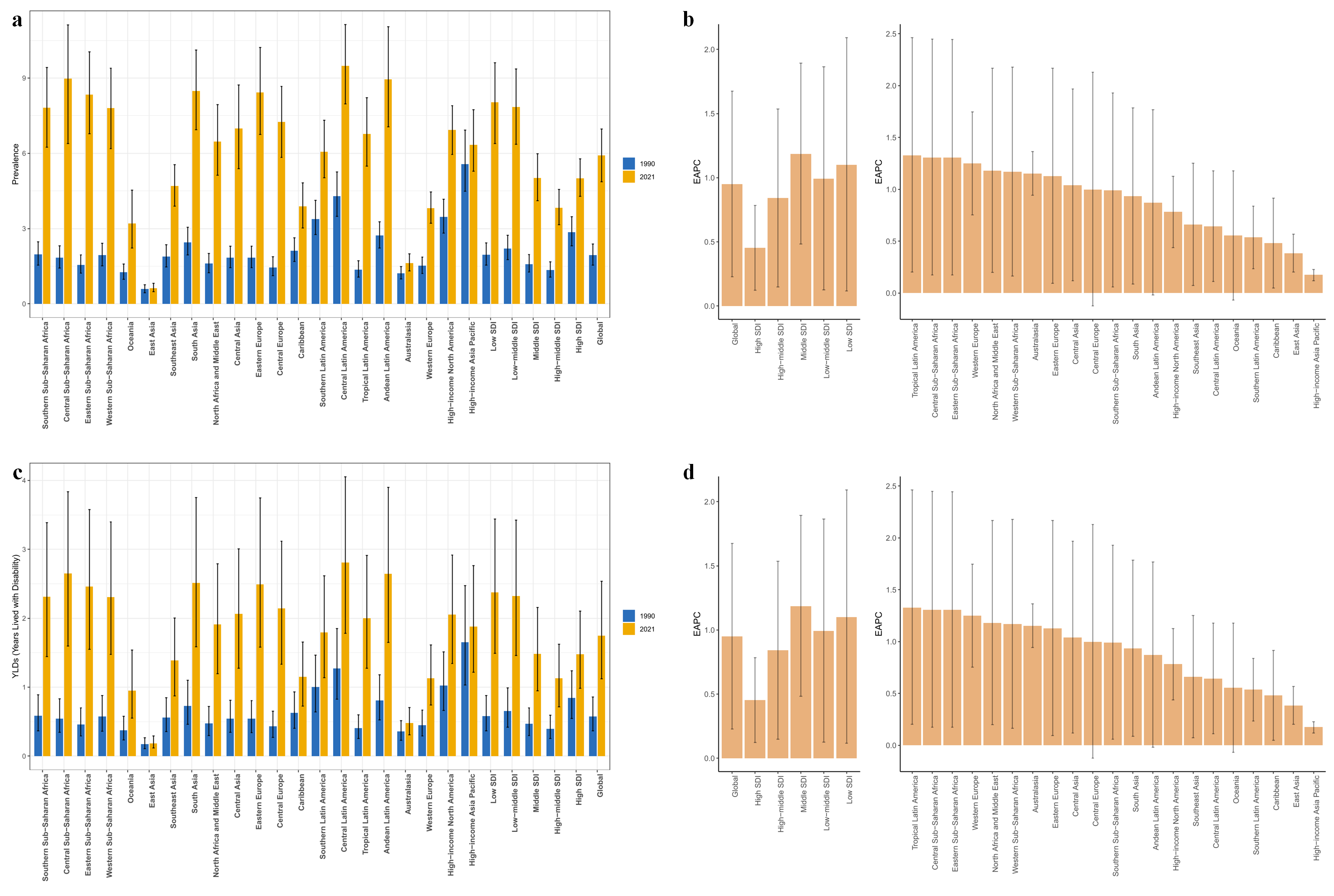

Supplement: Supplementary file 1 — Figure S1: Correlation between ASR of Guillain‐Barré syndrome and SDI at the national and regional levels in 2021. (a, b) Age‐standardized prevalence rate; (c, d) Age‐standardized YLDs rate. [file IID3-14-e70473-s002.png]

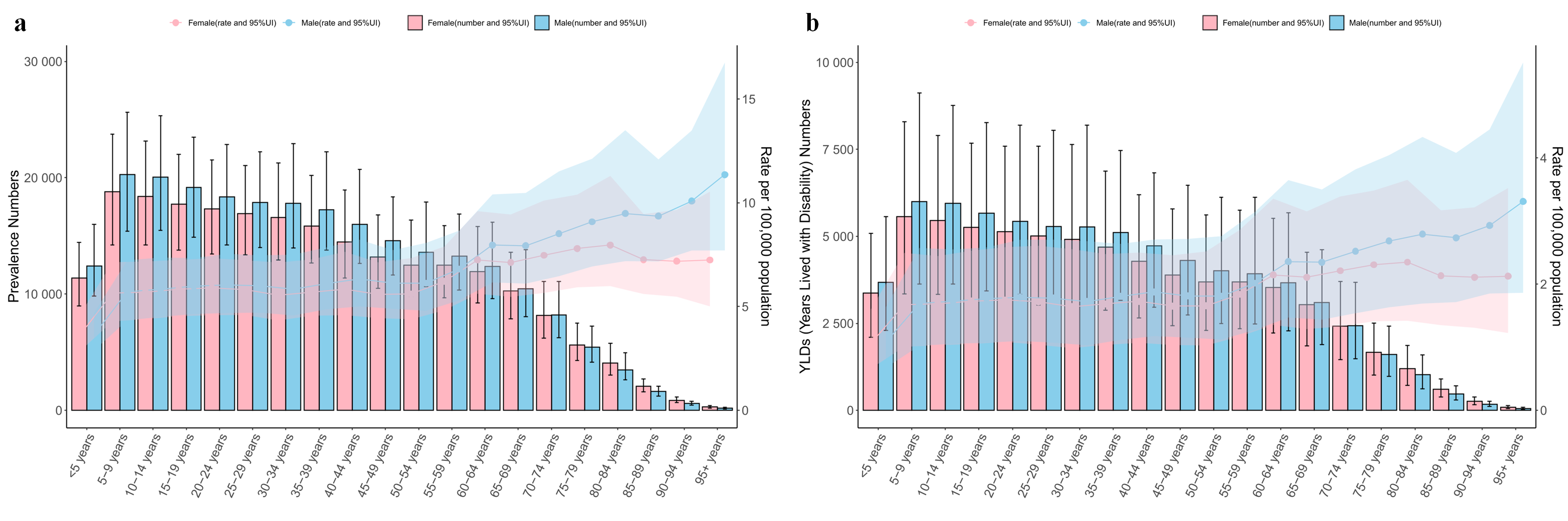

Supplement: Supplementary file 2 — Figure S2: Age distribution and trend of the Guillain‐Barré syndrome burden in 2021. (a) prevalent cases; (b) YLDs. [file IID3-14-e70473-s003.png]

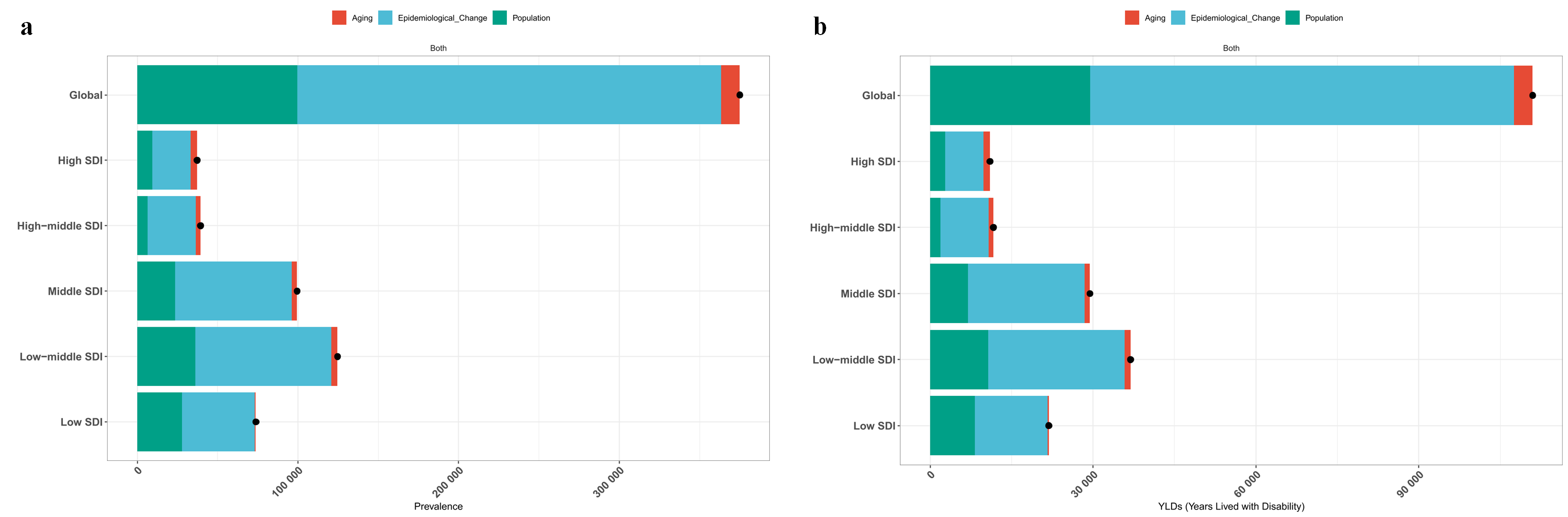

Supplement: Supplementary file 3 — Figure S3: Key drivers of Guillain‐Barré syndrome burden at global and SDI levels from 1990 to 2021: population growth, ageing, and epidemiological changes. The black dots represent the sum of contributions to changes in all three factors. (a) prevalent cases; (b) YLDs. [file IID3-14-e70473-s004.png]

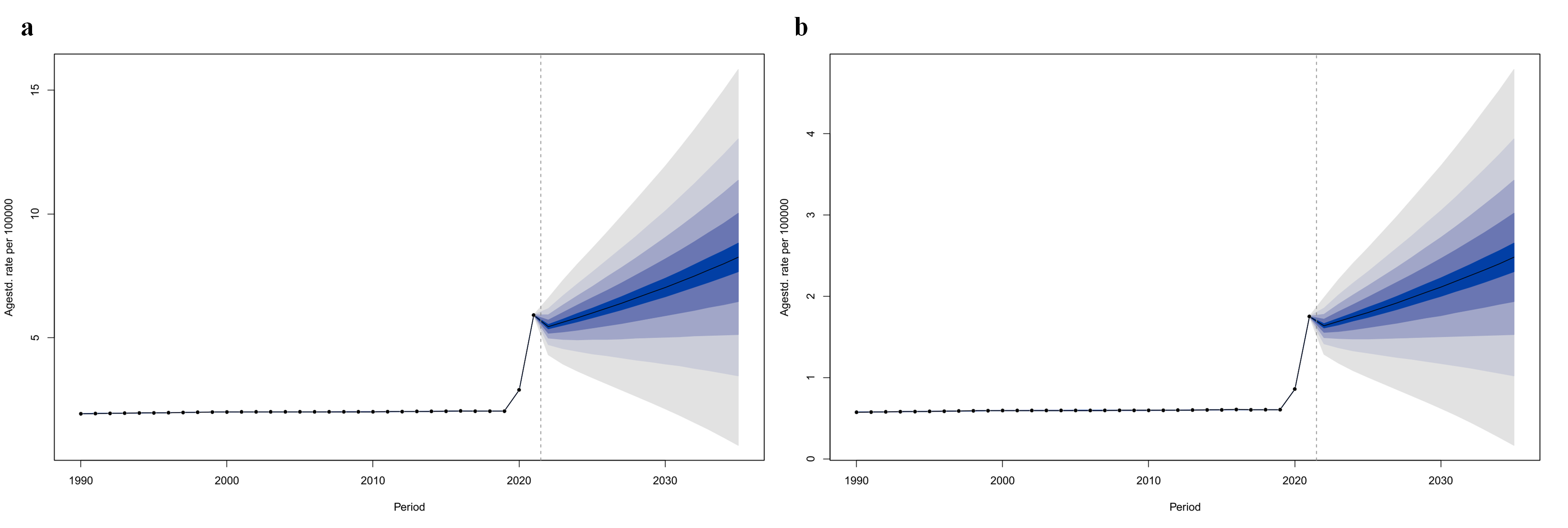

Supplement: Supplementary file 4 — Figure S4: Trends in the burden of Guillain‐Barré syndrome: observed rates (1990–2021) and predicted rates (2022–2035). The blue region shows the upper and lower limits of the 95% UI. (a) prevalent cases; (b) YLDs. [file IID3-14-e70473-s001.png]
