# Supplementary material for: Dynamic Changes and Future Trend Forecasts in the Global Burden of Guillain–Barré Syndrome: Analysis of 204 Countries and Regions From 1990 to 2021, Including the Impact of the COVID‐19 Pandemic
Source: Immun Inflamm Dis. 2026 Jun 17;14(6):e70473. doi: 10.1002/iid3.70473 (PMC13276001; doi:10.1002/iid3.70473)
Supplement: Supplementary file 5 — Supporting File [file IID3-14-e70473-s005.doc]

| **Table S1. The number of prevalent cases and YLDs of Guillain-Barré syndrome, along with corresponding ASR and temporal trends.** | | | | | | | | | | | |
| --- | --- | --- | --- | --- | --- | --- | --- | --- | --- | --- | --- |
| Characteristics | Prevalence(95% uncertainty interval) | | | | |  | YLDs(95% uncertainty interval) | | | | |
|  | Cases,1990 | Cases,2021 | ASPR,1990 | ASPR,2021 | EAPC 1990-2021 |  | Cases,1990 | Cases,2021 | ASYR,1990 | ASYR,2021 | EAPC 1990-2021 |
| Global | 96819.44(77059.09,121120.91) | 471850.04(389186.73,554144.88) | 1.93(1.54,2.38) | 5.91(4.87,6.97) | 0.95(0.53,1.37) |  | 28678.64(18066.62,43235.07) | 139639.04(90386.97,202386.86) | 0.57(0.37,0.86) | 1.75(1.12,2.54) | 0.95(0.53,1.36) |
| SDI level |  |  |  |  |  |  |  |  |  |  |  |
| High SDI | 26520.84(21387.59,32263.99) | 63822.67(55414.70,73636.15) | 2.86(2.31,3.48) | 4.99(4.29,5.78) | 0.45(0.22,0.67) |  | 7853.83(5086.03,11466.37) | 18887.92(12503.97,26432.13) | 0.85(0.55,1.24) | 1.48(0.98,2.10) | 0.45(0.22,0.67) |
| High-middle SDI | 14024.53(11038.52,17475.74) | 53487.23(44353.46,62347.70) | 1.34(1.07,1.67) | 3.82(3.16,4.56) | 0.84(0.43,1.24) |  | 4152.75(2638.98,6213.46) | 15808.64(10193.92,22788.12) | 0.40(0.25,0.59) | 1.13(0.72,1.63) | 0.83(0.43,1.24) |
| Middle SDI | 24992.31(19695.05,32023.89) | 124493.32(102524.74,147011.08) | 1.58(1.26,1.97) | 5.02(4.11,5.98) | 1.18(0.78,1.59) |  | 7403.11(4572.51,11291.46) | 36831.51(23568.54,53507.01) | 0.47(0.30,0.70) | 1.48(0.95,2.15) | 1.18(0.78,1.59) |
| Low-middle SDI | 22668.66(17726.75,29182.22) | 147339.87(118903.18,177566.94) | 2.21(1.76,2.73) | 7.84(6.36,9.36) | 0.99(0.51,1.48) |  | 6716.37(4104.68,10246.80) | 43632.85(27476.79,64257.03) | 0.65(0.42,0.99) | 2.32(1.46,3.42) | 0.99(0.51,1.48) |
| Low SDI | 8532.66(6619.54,11166.41) | 82360.00(65095.99,99251.15) | 1.95(1.54,2.43) | 8.03(6.39,9.60) | 1.11(0.54,1.67) |  | 2528.75(1532.07,3870.08) | 24375.51(15134.83,35235.88) | 0.58(0.37,0.88) | 2.37(1.49,3.44) | 1.10(0.54,1.67) |
| GBD Region |  |  |  |  |  |  |  |  |  |  |  |
| Andean Latin America | 847.76(694.58,1043.67) | 5868.36(4613.92,7275.88) | 2.72(2.23,3.27) | 8.94(7.05,11.05) | 0.88(0.31,1.46) |  | 251.04(160.19,365.65) | 1735.78(1082.23,2571.00) | 0.81(0.53,1.18) | 2.64(1.65,3.90) | 0.88(0.30,1.46) |
| Australasia | 254.70(207.47,312.98) | 591.45(465.62,736.57) | 1.21(0.99,1.49) | 1.62(1.31,2.00) | 1.15(0.99,1.31) |  | 75.36(47.72,108.38) | 175.06(111.46,259.00) | 0.36(0.23,0.51) | 0.48(0.31,0.70) | 1.15(0.99,1.32) |
| Caribbean | 697.81(550.57,891.95) | 1877.29(1471.36,2335.32) | 2.12(1.69,2.63) | 3.88(3.03,4.82) | 0.48(0.15,0.82) |  | 206.65(131.85,310.88) | 555.52(350.53,797.67) | 0.63(0.40,0.93) | 1.15(0.73,1.65) | 0.48(0.15,0.82) |
| Central Asia | 1168.87(903.70,1478.29) | 6603.30(5098.77,8253.53) | 1.83(1.45,2.30) | 6.98(5.39,8.72) | 1.03(0.52,1.55) |  | 346.29(217.13,527.70) | 1952.90(1204.81,2852.55) | 0.54(0.35,0.81) | 2.06(1.27,3.01) | 1.03(0.51,1.55) |
| Central Europe | 1892.65(1460.47,2411.02) | 9278.15(7595.61,11047.15) | 1.45(1.12,1.87) | 7.24(5.83,8.66) | 0.99(0.34,1.65) |  | 560.45(355.51,850.00) | 2742.09(1719.34,4000.68) | 0.43(0.27,0.65) | 2.14(1.33,3.12) | 0.99(0.34,1.65) |
| Central Latin America | 6427.60(5135.21,8201.40) | 23965.67(20088.83,28097.91) | 4.30(3.49,5.25) | 9.49(7.97,11.13) | 0.65(0.29,1.01) |  | 1904.12(1154.28,2866.57) | 7094.64(4484.59,10211.42) | 1.27(0.83,1.85) | 2.81(1.78,4.05) | 0.65(0.29,1.01) |
| Central Sub-Saharan Africa | 871.52(670.06,1167.29) | 11132.44(7867.86,13861.93) | 1.84(1.43,2.31) | 8.97(6.38,11.12) | 1.31(0.65,1.98) |  | 258.29(157.66,392.68) | 3290.57(1973.56,4828.68) | 0.54(0.35,0.83) | 2.65(1.60,3.84) | 1.31(0.65,1.98) |
| East Asia | 6716.73(4916.49,9069.62) | 9893.83(7377.67,12857.34) | 0.59(0.44,0.76) | 0.63(0.48,0.82) | 0.39(0.25,0.53) |  | 1988.58(1197.07,3103.21) | 2928.59(1797.87,4469.28) | 0.17(0.11,0.27) | 0.19(0.12,0.29) | 0.39(0.25,0.53) |
| Eastern Europe | 4334.43(3389.82,5405.87) | 19125.20(15419.86,23116.48) | 1.83(1.44,2.30) | 8.43(6.75,10.21) | 1.12(0.54,1.71) |  | 1283.67(818.31,1917.95) | 5643.45(3600.67,8477.53) | 0.54(0.34,0.81) | 2.49(1.58,3.75) | 1.12(0.54,1.70) |
| Eastern Sub-Saharan Africa | 2665.80(2051.74,3563.45) | 32607.04(26109.82,39433.77) | 1.55(1.22,1.94) | 8.33(6.78,10.04) | 1.31(0.64,2.00) |  | 790.28(479.53,1225.15) | 9639.70(6075.86,14104.76) | 0.46(0.29,0.70) | 2.46(1.55,3.58) | 1.31(0.63,1.99) |
| High-income Asia Pacific | 9717.76(7751.02,12119.08) | 11629.67(9758.69,13834.10) | 5.57(4.49,6.93) | 6.34(5.28,7.74) | 0.17(-0.00,0.34) |  | 2878.98(1826.16,4306.17) | 3442.83(2267.49,4991.44) | 1.65(1.03,2.47) | 1.88(1.22,2.77) | 0.17(-0.01,0.34) |
| High-income North America | 10608.45(8653.01,12907.43) | 31481.10(27534.95,35799.87) | 3.46(2.82,4.17) | 6.93(5.96,7.90) | 0.77(0.57,0.98) |  | 3141.36(2030.97,4647.15) | 9317.39(6134.60,12990.48) | 1.02(0.66,1.51) | 2.05(1.34,2.92) | 0.77(0.57,0.97) |
| North Africa and Middle East | 4591.33(3540.90,6010.47) | 38856.50(30710.18,47881.12) | 1.60(1.24,2.01) | 6.47(5.13,7.94) | 1.18(0.64,1.73) |  | 1359.96(846.94,2113.56) | 11496.88(7168.11,16760.54) | 0.47(0.30,0.72) | 1.91(1.19,2.79) | 1.18(0.63,1.72) |
| Oceania | 73.55(55.29,98.49) | 430.51(291.28,612.64) | 1.26(0.98,1.59) | 3.21(2.22,4.54) | 0.55(0.20,0.91) |  | 21.79(13.36,33.97) | 127.39(73.22,208.35) | 0.37(0.23,0.58) | 0.95(0.55,1.54) | 0.55(0.20,0.91) |
| South Asia | 23751.02(18471.24,30582.03) | 155149.88(126682.37,185327.56) | 2.45(1.96,3.05) | 8.48(6.93,10.11) | 0.93(0.46,1.40) |  | 7036.84(4328.32,10761.44) | 45944.74(28849.28,68526.23) | 0.73(0.46,1.10) | 2.51(1.58,3.75) | 0.93(0.46,1.40) |
| Southeast Asia | 7944.11(6105.58,10343.51) | 32889.16(27153.93,39008.35) | 1.89(1.47,2.36) | 4.69(3.89,5.55) | 0.66(0.32,1.00) |  | 2353.86(1458.03,3601.89) | 9734.51(6142.54,14040.96) | 0.56(0.35,0.85) | 1.39(0.88,2.00) | 0.66(0.32,1.00) |
| Southern Latin America | 1649.68(1352.75,2020.76) | 4314.34(3603.67,5150.46) | 3.38(2.76,4.13) | 6.05(5.02,7.31) | 0.53(0.30,0.77) |  | 488.59(314.68,716.19) | 1277.96(808.66,1849.21) | 1.00(0.64,1.47) | 1.79(1.14,2.62) | 0.53(0.30,0.77) |
| Southern Sub-Saharan Africa | 926.80(713.18,1212.06) | 6183.18(4925.00,7480.45) | 1.97(1.55,2.47) | 7.82(6.24,9.42) | 0.99(0.43,1.56) |  | 274.69(169.33,427.21) | 1829.78(1136.42,2675.93) | 0.58(0.37,0.89) | 2.31(1.45,3.39) | 0.99(0.43,1.56) |
| Tropical Latin America | 1703.49(1324.10,2168.17) | 15981.77(13016.04,19371.23) | 1.36(1.07,1.72) | 6.76(5.48,8.21) | 1.34(0.66,2.02) |  | 504.22(318.58,759.53) | 4723.73(3007.54,6862.95) | 0.40(0.26,0.60) | 2.00(1.27,2.91) | 1.34(0.66,2.02) |
| Western Europe | 6701.63(5317.65,8347.37) | 19783.42(16853.79,23082.29) | 1.52(1.22,1.87) | 3.81(3.21,4.46) | 1.24(0.86,1.62) |  | 1983.24(1256.30,2950.99) | 5856.03(3888.88,8259.78) | 0.45(0.29,0.67) | 1.13(0.74,1.61) | 1.24(0.86,1.62) |
| Western Sub-Saharan Africa | 3273.74(2534.30,4306.68) | 34207.80(26980.88,41334.82) | 1.94(1.52,2.42) | 7.79(6.18,9.38) | 1.18(0.58,1.78) |  | 970.37(591.01,1477.47) | 10129.51(6418.81,14958.34) | 0.57(0.36,0.88) | 2.31(1.48,3.40) | 1.18(0.58,1.78) |
| National level |  |  |  |  |  |  |  |  |  |  |  |
| Afghanistan | 141.57(108.94,181.49) | 2197.56(1375.96,2824.84) | 1.60(1.24,2.02) | 8.10(5.23,10.34) | 1.39(0.74,2.04) |  | 41.93(26.48,63.51) | 651.68(342.18,1027.36) | 0.47(0.30,0.73) | 2.40(1.25,3.79) | 1.39(0.74,2.04) |
| Albania | 43.66(33.34,56.51) | 312.88(218.12,394.43) | 1.42(1.11,1.80) | 10.78(7.46,13.54) | 1.50(0.72,2.29) |  | 12.93(8.02,19.92) | 91.30(53.19,141.02) | 0.42(0.27,0.63) | 3.16(1.83,4.78) | 1.50(0.71,2.28) |
| Algeria | 338.70(258.82,449.41) | 1287.42(937.65,1722.39) | 1.60(1.24,2.02) | 2.99(2.20,3.97) | 0.50(0.25,0.75) |  | 100.34(62.23,155.50) | 381.22(231.12,581.81) | 0.47(0.30,0.73) | 0.89(0.54,1.35) | 0.50(0.25,0.75) |
| American Samoa | 0.55(0.41,0.73) | 0.75(0.58,0.95) | 1.26(0.98,1.59) | 1.51(1.17,1.88) | 0.18(0.07,0.29) |  | 0.16(0.10,0.25) | 0.22(0.14,0.33) | 0.37(0.23,0.58) | 0.45(0.28,0.68) | 0.18(0.07,0.29) |
| Andorra | 1.02(0.79,1.30) | 5.78(4.49,7.24) | 1.78(1.39,2.23) | 6.01(4.55,7.70) | 0.99(0.46,1.53) |  | 0.30(0.19,0.46) | 1.71(1.10,2.52) | 0.53(0.34,0.81) | 1.78(1.11,2.65) | 0.99(0.46,1.53) |
| Angola | 162.36(124.55,218.26) | 2742.42(1710.88,3598.23) | 1.84(1.43,2.31) | 9.40(5.86,12.29) | 1.18(0.53,1.83) |  | 48.12(29.48,73.42) | 811.63(412.61,1236.66) | 0.55(0.35,0.83) | 2.78(1.43,4.20) | 1.18(0.53,1.83) |
| Antigua and Barbuda | 1.21(0.96,1.54) | 3.01(2.32,3.73) | 2.10(1.67,2.62) | 3.23(2.51,3.99) | 0.43(0.13,0.73) |  | 0.36(0.23,0.54) | 0.89(0.54,1.33) | 0.62(0.40,0.93) | 0.96(0.59,1.43) | 0.43(0.13,0.73) |
| Argentina | 1233.59(990.23,1533.55) | 3141.58(2551.02,3898.62) | 3.71(2.96,4.59) | 6.63(5.35,8.30) | 0.47(0.26,0.69) |  | 365.47(232.03,542.19) | 930.59(586.36,1359.65) | 1.10(0.70,1.63) | 1.96(1.24,2.89) | 0.47(0.26,0.69) |
| Armenia | 60.05(46.45,75.64) | 307.79(204.77,396.34) | 1.83(1.45,2.30) | 9.58(6.35,12.34) | 1.32(0.68,1.96) |  | 17.79(11.20,26.93) | 90.73(50.33,137.31) | 0.54(0.35,0.81) | 2.82(1.57,4.24) | 1.32(0.68,1.96) |
| Australia | 173.64(138.97,217.81) | 462.86(358.54,584.99) | 1.00(0.81,1.25) | 1.52(1.20,1.91) | 1.63(1.44,1.81) |  | 51.38(32.28,74.31) | 137.03(85.96,205.91) | 0.30(0.19,0.43) | 0.45(0.29,0.67) | 1.63(1.44,1.81) |
| Austria | 217.87(183.93,259.07) | 663.31(566.13,786.31) | 2.31(1.97,2.74) | 5.62(4.72,6.75) | 1.54(1.13,1.95) |  | 64.47(41.83,91.71) | 196.24(125.71,279.65) | 0.68(0.44,0.96) | 1.66(1.04,2.39) | 1.54(1.13,1.95) |
| Azerbaijan | 123.95(95.48,156.27) | 966.52(569.69,1325.17) | 1.83(1.45,2.30) | 8.96(5.28,12.28) | 1.12(0.52,1.71) |  | 36.72(23.01,55.88) | 285.82(151.28,457.00) | 0.54(0.35,0.81) | 2.65(1.41,4.22) | 1.11(0.52,1.71) |
| Bahrain | 6.81(5.06,9.31) | 98.65(67.90,135.57) | 1.61(1.25,2.04) | 6.54(4.55,8.94) | 1.06(0.52,1.59) |  | 2.02(1.23,3.18) | 29.17(16.91,44.41) | 0.48(0.30,0.73) | 1.93(1.13,2.93) | 1.06(0.52,1.59) |
| Bangladesh | 2067.15(1613.30,2697.33) | 13758.79(9722.64,18423.62) | 2.23(1.79,2.79) | 8.40(5.95,11.23) | 1.03(0.53,1.54) |  | 612.66(379.78,921.14) | 4077.09(2307.98,6279.25) | 0.66(0.42,0.98) | 2.49(1.41,3.82) | 1.03(0.53,1.54) |
| Barbados | 5.35(4.25,6.72) | 9.39(7.47,11.46) | 2.10(1.67,2.62) | 2.78(2.25,3.35) | 0.23(-0.02,0.49) |  | 1.58(1.00,2.36) | 2.78(1.77,4.13) | 0.62(0.40,0.93) | 0.82(0.53,1.21) | 0.23(-0.02,0.49) |
| Belarus | 184.21(142.77,232.60) | 660.47(353.76,1057.47) | 1.68(1.31,2.14) | 6.44(3.49,10.34) | 1.01(0.49,1.53) |  | 54.55(34.13,82.27) | 195.63(95.16,348.99) | 0.50(0.31,0.76) | 1.91(0.93,3.42) | 1.01(0.49,1.53) |
| Belgium | 205.33(157.20,260.52) | 580.17(465.54,705.12) | 1.76(1.37,2.21) | 4.36(3.48,5.33) | 0.72(0.30,1.15) |  | 60.80(37.96,92.69) | 171.71(110.79,248.65) | 0.52(0.33,0.79) | 1.29(0.83,1.87) | 0.72(0.30,1.15) |
| Belize | 3.54(2.78,4.72) | 21.51(13.11,31.44) | 2.13(1.70,2.65) | 5.19(3.21,7.47) | 0.77(0.37,1.17) |  | 1.05(0.65,1.57) | 6.37(3.24,10.30) | 0.63(0.41,0.94) | 1.54(0.78,2.45) | 0.77(0.37,1.17) |
| Benin | 76.51(58.94,103.27) | 504.14(337.74,735.40) | 1.83(1.42,2.31) | 4.22(2.86,6.12) | 0.62(0.24,1.00) |  | 22.68(13.74,34.58) | 149.32(84.41,250.13) | 0.54(0.34,0.83) | 1.25(0.71,2.10) | 0.62(0.24,1.00) |
| Bermuda | 1.26(0.99,1.57) | 2.30(1.89,2.78) | 2.11(1.67,2.62) | 3.04(2.53,3.66) | 0.24(-0.03,0.51) |  | 0.37(0.24,0.55) | 0.68(0.44,1.00) | 0.62(0.40,0.93) | 0.90(0.58,1.31) | 0.24(-0.03,0.51) |
| Bhutan | 11.81(9.09,15.72) | 17.81(14.36,22.05) | 2.25(1.81,2.81) | 2.45(2.02,3.02) | 0.06(0.02,0.09) |  | 3.50(2.20,5.26) | 5.27(3.37,7.78) | 0.67(0.42,0.99) | 0.73(0.47,1.07) | 0.06(0.02,0.09) |
| Bolivarian Republic of Venezuela | 665.48(524.31,860.81) | 2160.13(1767.41,2598.44) | 3.89(3.15,4.81) | 7.91(6.50,9.49) | 0.56(0.24,0.89) |  | 197.16(120.84,296.96) | 639.52(402.96,941.39) | 1.15(0.75,1.68) | 2.34(1.46,3.47) | 0.56(0.24,0.89) |
| Bosnia and Herzegovina | 59.47(45.14,76.24) | 317.51(225.44,414.77) | 1.34(1.05,1.71) | 8.68(6.08,11.34) | 1.26(0.46,2.06) |  | 17.60(11.03,27.24) | 93.69(53.67,142.86) | 0.40(0.25,0.61) | 2.56(1.47,3.91) | 1.26(0.46,2.06) |
| Botswana | 20.95(16.13,28.12) | 149.95(86.13,216.23) | 1.82(1.42,2.29) | 6.38(3.73,9.18) | 0.74(0.23,1.25) |  | 6.21(3.83,9.47) | 44.35(22.50,73.76) | 0.54(0.34,0.82) | 1.89(0.97,3.13) | 0.74(0.23,1.25) |
| Brazil | 1662.80(1291.20,2118.26) | 15483.04(12620.51,18827.52) | 1.37(1.07,1.72) | 6.75(5.47,8.21) | 1.34(0.67,2.03) |  | 492.18(311.24,741.29) | 4575.90(2903.20,6624.94) | 0.40(0.26,0.60) | 2.00(1.27,2.90) | 1.34(0.66,2.03) |
| Brunei Darussalam | 15.15(11.75,19.58) | 28.17(22.68,35.72) | 5.88(4.75,7.34) | 6.11(5.00,7.56) | 0.03(-0.11,0.17) |  | 4.49(2.72,6.94) | 8.35(5.24,12.69) | 1.74(1.10,2.62) | 1.81(1.14,2.73) | 0.03(-0.11,0.17) |
| Bulgaria | 135.24(105.42,171.37) | 662.64(404.07,878.31) | 1.42(1.11,1.80) | 8.67(5.30,11.68) | 1.22(0.52,1.92) |  | 40.04(25.20,59.99) | 196.46(99.92,311.36) | 0.42(0.27,0.63) | 2.57(1.32,4.12) | 1.22(0.53,1.93) |
| Burkina Faso | 152.14(117.34,203.82) | 1751.89(1208.57,2258.62) | 1.83(1.42,2.30) | 8.70(6.01,11.24) | 1.22(0.59,1.86) |  | 45.10(27.43,68.06) | 518.67(293.93,787.47) | 0.54(0.34,0.83) | 2.57(1.46,3.87) | 1.22(0.59,1.86) |
| Burundi | 75.21(57.56,98.84) | 350.93(264.77,447.38) | 1.51(1.18,1.89) | 2.95(2.26,3.71) | 0.44(0.09,0.79) |  | 22.30(13.65,35.07) | 103.96(65.81,156.81) | 0.45(0.28,0.68) | 0.87(0.55,1.32) | 0.44(0.09,0.79) |
| Cambodia | 156.57(120.66,205.73) | 492.16(355.07,630.93) | 1.78(1.41,2.23) | 2.96(2.17,3.77) | 0.29(0.09,0.50) |  | 46.41(28.20,71.91) | 145.71(87.12,217.30) | 0.53(0.33,0.79) | 0.88(0.53,1.31) | 0.29(0.09,0.50) |
| Cameroon | 166.22(127.88,221.33) | 1934.45(642.06,2939.61) | 1.84(1.43,2.31) | 6.73(2.27,10.18) | 1.04(0.50,1.59) |  | 49.26(30.00,74.52) | 574.24(171.85,1002.92) | 0.54(0.35,0.83) | 2.00(0.63,3.46) | 1.04(0.50,1.59) |
| Canada | 573.53(449.02,711.54) | 1972.98(1619.17,2401.53) | 1.92(1.52,2.35) | 4.01(3.33,4.80) | 1.25(1.04,1.47) |  | 169.59(110.17,247.56) | 584.09(368.58,870.88) | 0.57(0.37,0.83) | 1.19(0.75,1.74) | 1.26(1.04,1.47) |
| Central African Republic | 43.40(33.29,57.92) | 307.55(218.11,434.87) | 1.83(1.42,2.30) | 6.17(4.39,8.67) | 1.14(0.58,1.70) |  | 12.86(7.84,19.55) | 91.17(52.14,146.69) | 0.54(0.34,0.83) | 1.83(1.06,2.95) | 1.14(0.58,1.71) |
| Chad | 96.08(74.08,128.44) | 1004.62(627.66,1522.30) | 1.83(1.42,2.31) | 6.60(4.18,9.99) | 1.17(0.60,1.74) |  | 28.48(17.37,42.97) | 297.73(164.36,507.52) | 0.54(0.34,0.83) | 1.96(1.07,3.35) | 1.17(0.60,1.75) |
| Chile | 303.46(255.16,367.73) | 970.56(802.34,1152.29) | 2.51(2.12,3.01) | 4.79(3.96,5.66) | 0.92(0.63,1.21) |  | 89.76(56.08,128.84) | 287.49(184.00,415.73) | 0.74(0.47,1.06) | 1.42(0.91,2.05) | 0.92(0.63,1.22) |
| China | 6398.35(4668.46,8660.46) | 9386.71(6966.98,12260.16) | 0.58(0.43,0.75) | 0.62(0.47,0.80) | 0.39(0.24,0.53) |  | 1894.33(1138.62,2960.49) | 2778.53(1697.73,4245.56) | 0.17(0.11,0.26) | 0.18(0.11,0.29) | 0.39(0.24,0.53) |
| Colombia | 1146.74(905.52,1465.25) | 3982.01(3145.75,4903.50) | 3.90(3.15,4.81) | 8.03(6.35,9.90) | 0.55(0.22,0.88) |  | 339.72(209.07,506.66) | 1179.60(731.80,1735.10) | 1.15(0.75,1.68) | 2.38(1.47,3.49) | 0.55(0.22,0.88) |
| Commonwealth of the Bahamas | 4.88(3.81,6.30) | 16.37(11.72,21.79) | 2.10(1.67,2.62) | 4.12(2.98,5.50) | 0.51(0.17,0.85) |  | 1.45(0.91,2.22) | 4.85(2.83,7.35) | 0.62(0.40,0.93) | 1.22(0.72,1.87) | 0.51(0.17,0.85) |
| Comoros | 6.31(4.83,8.27) | 63.78(47.72,83.94) | 1.52(1.19,1.89) | 8.86(6.71,11.60) | 1.18(0.48,1.89) |  | 1.87(1.14,2.92) | 18.88(11.13,27.77) | 0.45(0.29,0.68) | 2.62(1.55,3.84) | 1.18(0.48,1.89) |
| Congo | 38.34(29.58,51.32) | 353.26(270.62,451.82) | 1.83(1.42,2.30) | 6.95(5.35,8.79) | 1.12(0.56,1.70) |  | 11.36(6.99,17.26) | 104.58(63.44,153.12) | 0.54(0.34,0.83) | 2.06(1.27,3.01) | 1.12(0.56,1.70) |
| Cook Islands | 0.22(0.17,0.29) | 0.44(0.33,0.55) | 1.26(0.98,1.59) | 2.35(1.82,2.98) | 0.43(0.18,0.67) |  | 0.07(0.04,0.10) | 0.13(0.08,0.19) | 0.37(0.23,0.58) | 0.69(0.43,1.01) | 0.43(0.18,0.67) |
| Costa Rica | 109.23(86.04,140.16) | 368.63(279.53,469.91) | 3.90(3.16,4.82) | 7.61(5.79,9.80) | 0.46(0.15,0.78) |  | 32.36(19.95,48.48) | 109.10(69.18,168.00) | 1.16(0.75,1.68) | 2.25(1.43,3.44) | 0.46(0.15,0.78) |
| Croatia | 72.59(56.05,92.50) | 313.39(248.76,405.10) | 1.41(1.10,1.79) | 6.56(5.15,8.50) | 1.01(0.42,1.61) |  | 21.49(13.54,32.17) | 92.79(58.68,135.96) | 0.42(0.26,0.63) | 1.94(1.21,2.89) | 1.01(0.42,1.61) |
| Cuba | 223.21(176.41,278.89) | 391.27(318.39,474.90) | 2.13(1.69,2.64) | 3.07(2.52,3.73) | 0.28(0.01,0.55) |  | 66.09(41.91,99.51) | 115.80(74.78,170.45) | 0.63(0.41,0.93) | 0.91(0.58,1.34) | 0.28(0.01,0.55) |
| Cyprus | 14.16(11.02,17.84) | 44.67(37.27,53.73) | 1.76(1.38,2.21) | 2.91(2.44,3.45) | 0.30(0.00,0.61) |  | 4.19(2.64,6.39) | 13.22(8.46,19.10) | 0.52(0.33,0.79) | 0.86(0.56,1.23) | 0.30(0.00,0.61) |
| Czech Republic | 155.06(120.30,196.09) | 1081.85(751.38,1370.94) | 1.41(1.10,1.79) | 9.09(6.31,11.62) | 1.27(0.55,1.99) |  | 45.91(29.01,68.38) | 317.48(195.90,472.38) | 0.42(0.27,0.63) | 2.68(1.63,4.01) | 1.26(0.55,1.98) |
| Democratic People's Republic of Korea | 159.29(119.55,208.33) | 230.01(172.79,294.96) | 0.82(0.63,1.05) | 0.84(0.65,1.08) | 0.02(-0.07,0.11) |  | 47.14(28.91,72.40) | 68.06(42.77,102.54) | 0.24(0.15,0.37) | 0.25(0.15,0.38) | 0.02(-0.07,0.11) |
| Democratic Republic of the Congo | 604.44(463.74,809.72) | 7523.69(4984.59,9510.51) | 1.84(1.43,2.31) | 9.22(6.10,11.67) | 1.37(0.69,2.06) |  | 179.14(109.17,271.95) | 2222.22(1292.29,3305.67) | 0.54(0.35,0.83) | 2.72(1.59,4.02) | 1.37(0.69,2.05) |
| Denmark | 53.46(43.01,66.30) | 164.09(132.96,198.51) | 0.91(0.74,1.14) | 2.42(1.98,2.95) | 1.71(1.37,2.05) |  | 15.81(9.94,22.62) | 48.56(30.30,71.44) | 0.27(0.17,0.39) | 0.72(0.46,1.06) | 1.71(1.37,2.05) |
| Djibouti | 5.59(4.23,7.40) | 71.90(44.65,107.67) | 1.53(1.19,1.91) | 5.95(3.74,8.87) | 1.21(0.60,1.82) |  | 1.66(1.02,2.63) | 21.26(12.02,33.87) | 0.45(0.29,0.68) | 1.76(1.00,2.83) | 1.21(0.60,1.82) |
| Dominica | 1.45(1.15,1.85) | 2.03(1.61,2.52) | 2.10(1.67,2.62) | 2.87(2.30,3.58) | 0.47(0.13,0.83) |  | 0.43(0.27,0.64) | 0.60(0.38,0.87) | 0.62(0.40,0.93) | 0.85(0.54,1.24) | 0.47(0.12,0.82) |
| Dominican Republic | 134.36(104.65,175.25) | 533.13(332.40,730.43) | 2.12(1.69,2.64) | 4.89(3.09,6.69) | 0.76(0.36,1.16) |  | 39.80(24.91,60.38) | 157.72(86.34,251.42) | 0.63(0.41,0.93) | 1.45(0.80,2.31) | 0.76(0.36,1.16) |
| Ecuador | 227.50(194.44,277.11) | 1523.90(1138.25,1895.39) | 2.82(2.42,3.34) | 8.51(6.36,10.56) | 0.38(-0.25,1.01) |  | 67.31(43.42,95.22) | 451.22(275.70,682.92) | 0.83(0.54,1.17) | 2.52(1.54,3.81) | 0.37(-0.25,1.01) |
| Egypt | 752.46(576.63,986.72) | 7190.81(2539.36,10619.20) | 1.60(1.24,2.02) | 7.29(2.59,10.71) | 1.35(0.73,1.97) |  | 222.84(138.61,349.97) | 2128.44(662.55,3578.78) | 0.47(0.30,0.73) | 2.16(0.68,3.63) | 1.35(0.73,1.97) |
| El Salvador | 169.05(132.85,215.63) | 401.66(312.37,499.16) | 3.49(2.84,4.33) | 6.31(4.91,7.83) | 0.62(0.32,0.92) |  | 50.08(31.04,74.12) | 118.87(73.06,178.23) | 1.03(0.66,1.50) | 1.87(1.14,2.79) | 0.62(0.32,0.92) |
| Equatorial Guinea | 6.75(5.21,8.94) | 89.80(66.55,115.99) | 1.82(1.42,2.29) | 6.48(4.91,8.22) | 1.30(0.69,1.92) |  | 2.00(1.22,3.02) | 26.63(15.55,39.93) | 0.54(0.34,0.82) | 1.92(1.14,2.88) | 1.30(0.69,1.92) |
| Eritrea | 45.80(34.71,60.52) | 208.77(155.53,274.41) | 1.50(1.17,1.89) | 3.42(2.57,4.48) | 0.50(0.11,0.90) |  | 13.58(8.25,21.37) | 61.72(38.54,94.86) | 0.45(0.28,0.68) | 1.01(0.64,1.53) | 0.50(0.10,0.89) |
| Estonia | 27.95(21.79,35.15) | 61.73(49.40,81.47) | 1.68(1.31,2.14) | 4.19(3.28,5.59) | 0.56(0.20,0.91) |  | 8.28(5.23,12.37) | 18.28(11.33,29.17) | 0.50(0.31,0.76) | 1.24(0.77,2.01) | 0.56(0.20,0.91) |
| Ethiopia | 774.53(595.62,1044.93) | 9933.25(7883.60,11991.96) | 1.69(1.33,2.13) | 9.92(7.95,11.88) | 1.32(0.60,2.05) |  | 229.62(141.29,355.01) | 2934.84(1831.40,4302.48) | 0.50(0.31,0.76) | 2.92(1.84,4.28) | 1.32(0.59,2.04) |
| Federated States of Micronesia | 1.18(0.89,1.58) | 1.22(0.92,1.58) | 1.26(0.98,1.59) | 1.26(0.98,1.59) | 0.00(-0.08,0.09) |  | 0.35(0.21,0.54) | 0.36(0.22,0.57) | 0.37(0.23,0.58) | 0.37(0.23,0.58) | 0.00(-0.08,0.09) |
| Fiji | 8.58(6.40,11.42) | 27.18(17.69,36.13) | 1.26(0.98,1.59) | 2.98(1.96,3.96) | 0.50(0.17,0.83) |  | 2.54(1.56,3.99) | 8.04(4.81,12.37) | 0.37(0.23,0.58) | 0.88(0.53,1.35) | 0.50(0.17,0.83) |
| Finland | 59.68(48.48,73.31) | 116.13(99.20,138.77) | 1.09(0.89,1.35) | 1.82(1.54,2.16) | 0.71(0.47,0.95) |  | 17.65(11.25,25.42) | 34.35(22.42,48.30) | 0.32(0.21,0.46) | 0.54(0.35,0.77) | 0.71(0.47,0.95) |
| France | 1172.62(911.96,1473.07) | 2550.38(2053.74,3395.20) | 1.80(1.41,2.25) | 3.48(2.76,4.70) | 0.37(-0.08,0.81) |  | 347.16(222.14,516.88) | 754.51(474.00,1133.10) | 0.53(0.34,0.81) | 1.03(0.65,1.53) | 0.37(-0.08,0.81) |
| Gabon | 16.23(12.47,21.21) | 115.72(67.12,156.14) | 1.83(1.42,2.30) | 6.73(3.91,9.07) | 1.13(0.56,1.70) |  | 4.81(2.93,7.28) | 34.34(16.16,54.55) | 0.54(0.34,0.83) | 2.00(0.95,3.17) | 1.13(0.56,1.70) |
| Georgia | 104.89(82.03,130.62) | 356.47(188.93,478.01) | 1.83(1.45,2.30) | 9.02(4.78,12.08) | 0.90(0.29,1.51) |  | 31.06(19.84,46.76) | 104.39(48.28,167.58) | 0.54(0.35,0.81) | 2.65(1.23,4.22) | 0.89(0.29,1.50) |
| Germany | 1346.88(1019.75,1701.10) | 3579.01(3017.63,4253.30) | 1.47(1.14,1.85) | 3.32(2.81,3.89) | 0.88(0.58,1.19) |  | 398.37(248.44,606.15) | 1059.52(698.87,1552.13) | 0.44(0.27,0.65) | 0.98(0.64,1.42) | 0.89(0.58,1.19) |
| Ghana | 238.86(184.27,320.72) | 2267.18(1555.92,2902.53) | 1.84(1.43,2.31) | 7.14(4.96,9.10) | 1.08(0.51,1.65) |  | 70.79(43.36,107.95) | 671.92(387.59,999.67) | 0.54(0.35,0.83) | 2.11(1.22,3.17) | 1.08(0.51,1.65) |
| Greece | 123.16(96.33,157.61) | 413.49(348.05,501.21) | 1.07(0.84,1.37) | 3.29(2.77,3.90) | 1.93(1.58,2.27) |  | 36.42(22.57,53.79) | 122.42(79.66,178.16) | 0.32(0.20,0.46) | 0.97(0.64,1.39) | 1.93(1.58,2.27) |
| Greenland | 1.62(1.25,2.04) | 2.34(1.83,3.01) | 3.35(2.66,4.15) | 3.61(2.90,4.47) | 0.05(0.02,0.09) |  | 0.48(0.30,0.70) | 0.69(0.44,1.07) | 0.99(0.63,1.48) | 1.07(0.69,1.60) | 0.05(0.02,0.09) |
| Grenada | 1.75(1.40,2.26) | 3.19(2.46,4.04) | 2.11(1.67,2.62) | 3.02(2.36,3.83) | 0.31(0.03,0.59) |  | 0.52(0.33,0.77) | 0.94(0.59,1.42) | 0.62(0.40,0.93) | 0.89(0.56,1.34) | 0.31(0.03,0.58) |
| Guam | 1.57(1.17,2.08) | 7.87(5.57,11.02) | 1.26(0.99,1.59) | 4.88(3.43,6.87) | 1.05(0.53,1.57) |  | 0.47(0.28,0.73) | 2.32(1.31,3.68) | 0.37(0.24,0.58) | 1.44(0.81,2.30) | 1.04(0.52,1.56) |
| Guatemala | 309.72(234.84,414.73) | 1459.35(1069.22,1891.58) | 3.90(3.15,4.82) | 9.61(7.13,12.46) | 0.76(0.37,1.15) |  | 91.78(55.00,139.71) | 431.78(253.90,654.73) | 1.15(0.75,1.68) | 2.84(1.68,4.29) | 0.76(0.37,1.15) |
| Guinea | 98.22(76.25,129.07) | 1016.56(692.56,1363.59) | 1.84(1.43,2.31) | 8.46(5.82,11.26) | 1.31(0.66,1.97) |  | 29.11(17.88,43.81) | 301.16(174.97,481.62) | 0.54(0.35,0.83) | 2.50(1.47,3.93) | 1.31(0.66,1.96) |
| Guinea-Bissau | 15.86(12.21,21.44) | 111.09(74.70,162.76) | 1.84(1.42,2.31) | 6.01(4.12,8.78) | 1.20(0.62,1.78) |  | 4.70(2.87,7.12) | 32.92(17.98,53.16) | 0.54(0.34,0.83) | 1.78(0.99,2.87) | 1.20(0.62,1.78) |
| Guyana | 14.52(11.29,18.95) | 40.32(24.33,57.73) | 2.12(1.69,2.64) | 5.36(3.25,7.64) | 0.63(0.22,1.05) |  | 4.30(2.69,6.54) | 11.97(6.29,20.01) | 0.63(0.40,0.93) | 1.59(0.84,2.65) | 0.64(0.22,1.05) |
| Haiti | 120.99(94.38,158.89) | 481.74(287.79,695.05) | 2.11(1.68,2.63) | 4.01(2.43,5.72) | 0.54(0.20,0.89) |  | 35.84(22.29,53.98) | 142.51(75.31,231.92) | 0.63(0.40,0.93) | 1.19(0.63,1.91) | 0.54(0.20,0.89) |
| Honduras | 145.55(112.90,194.46) | 1036.22(773.41,1310.68) | 3.42(2.76,4.26) | 10.74(8.15,13.47) | 1.00(0.53,1.48) |  | 43.11(26.51,65.22) | 306.60(188.24,461.56) | 1.01(0.64,1.50) | 3.18(1.95,4.80) | 1.01(0.54,1.48) |
| Hungary | 159.20(124.32,202.07) | 726.14(501.20,952.94) | 1.41(1.10,1.79) | 6.74(4.67,8.87) | 1.01(0.41,1.63) |  | 47.14(29.76,70.39) | 215.04(129.50,334.79) | 0.42(0.27,0.63) | 2.00(1.19,3.09) | 1.01(0.41,1.63) |
| Iceland | 4.60(3.59,5.81) | 9.07(7.22,11.25) | 1.76(1.38,2.21) | 2.23(1.81,2.71) | 0.19(-0.07,0.44) |  | 1.36(0.86,2.08) | 2.69(1.74,3.99) | 0.52(0.33,0.80) | 0.66(0.43,0.98) | 0.19(-0.07,0.44) |
| India | 18878.73(14683.48,24189.23) | 120438.80(98025.57,144220.05) | 2.48(1.98,3.09) | 8.53(6.95,10.19) | 0.91(0.45,1.38) |  | 5592.90(3462.11,8547.46) | 35665.95(22496.02,52522.12) | 0.74(0.47,1.12) | 2.53(1.61,3.72) | 0.91(0.45,1.38) |
| Indonesia | 3322.07(2507.66,4348.59) | 17890.33(14541.73,21586.10) | 1.98(1.53,2.49) | 6.43(5.24,7.69) | 0.78(0.34,1.23) |  | 984.27(613.50,1520.75) | 5294.87(3292.03,7640.28) | 0.59(0.37,0.89) | 1.90(1.19,2.75) | 0.78(0.34,1.23) |
| Iraq | 240.61(183.27,321.82) | 4362.19(2922.38,5677.08) | 1.60(1.24,2.02) | 11.24(7.59,14.64) | 1.67(0.89,2.46) |  | 71.28(44.06,110.48) | 1286.01(728.68,1946.65) | 0.47(0.30,0.73) | 3.30(1.84,5.04) | 1.67(0.89,2.45) |
| Ireland | 65.52(51.35,82.36) | 187.31(150.53,237.50) | 1.76(1.38,2.21) | 3.41(2.70,4.33) | 0.49(0.15,0.84) |  | 19.40(12.28,29.38) | 55.40(35.16,83.73) | 0.52(0.33,0.79) | 1.01(0.64,1.52) | 0.49(0.15,0.84) |
| Islamic Republic of Iran | 680.33(518.70,906.01) | 5772.28(4656.85,7013.16) | 1.38(1.07,1.75) | 6.70(5.38,8.08) | 1.43(0.82,2.05) |  | 201.55(122.08,313.60) | 1708.43(1051.65,2449.88) | 0.41(0.25,0.62) | 1.98(1.23,2.83) | 1.43(0.82,2.05) |
| Israel | 85.35(67.19,109.14) | 384.51(318.10,462.09) | 1.76(1.37,2.20) | 3.88(3.18,4.68) | 0.56(0.18,0.94) |  | 25.28(16.09,38.39) | 113.79(73.51,163.79) | 0.52(0.33,0.79) | 1.15(0.74,1.66) | 0.56(0.18,0.94) |
| Italy | 1536.84(1224.49,1885.25) | 3296.20(2812.60,3812.11) | 2.23(1.82,2.70) | 4.25(3.59,5.00) | 0.64(0.23,1.06) |  | 454.90(293.61,673.89) | 975.82(638.05,1389.70) | 0.66(0.43,0.97) | 1.26(0.82,1.78) | 0.64(0.23,1.06) |
| Jamaica | 46.74(37.18,60.22) | 101.71(73.94,129.77) | 2.11(1.68,2.63) | 3.56(2.59,4.56) | 0.63(0.25,1.02) |  | 13.85(8.73,20.80) | 30.11(17.56,45.88) | 0.63(0.40,0.93) | 1.05(0.62,1.60) | 0.63(0.25,1.01) |
| Japan | 6896.12(5493.53,8510.82) | 8102.35(6882.09,9477.33) | 5.49(4.42,6.86) | 6.53(5.47,7.91) | 0.23(0.04,0.42) |  | 2042.66(1309.11,3021.38) | 2398.08(1586.89,3443.13) | 1.63(1.01,2.45) | 1.93(1.26,2.83) | 0.23(0.04,0.42) |
| Jordan | 47.81(36.11,64.52) | 927.36(646.64,1197.86) | 1.60(1.24,2.02) | 8.05(5.60,10.35) | 1.04(0.45,1.65) |  | 14.16(8.64,21.93) | 273.58(155.96,421.13) | 0.47(0.30,0.73) | 2.37(1.32,3.72) | 1.04(0.45,1.64) |
| Kazakhstan | 284.35(220.60,359.67) | 1127.37(676.35,1725.27) | 1.83(1.44,2.29) | 5.95(3.58,9.11) | 0.88(0.43,1.33) |  | 84.22(53.24,128.65) | 333.98(173.45,579.94) | 0.54(0.35,0.81) | 1.76(0.92,3.06) | 0.88(0.43,1.33) |
| Kenya | 360.58(275.12,486.34) | 4765.34(3733.66,5780.76) | 1.75(1.37,2.19) | 9.99(7.99,12.02) | 1.36(0.66,2.05) |  | 106.92(65.88,163.98) | 1405.46(882.14,2082.84) | 0.52(0.33,0.78) | 2.94(1.85,4.33) | 1.35(0.66,2.05) |
| Kingdom of Eswatini | 12.56(9.64,17.02) | 86.09(50.39,133.98) | 1.82(1.41,2.29) | 7.81(4.65,12.16) | 1.03(0.45,1.62) |  | 3.72(2.29,5.65) | 25.47(12.51,44.26) | 0.54(0.34,0.82) | 2.31(1.15,3.99) | 1.03(0.45,1.61) |
| Kiribati | 0.84(0.63,1.12) | 1.44(1.10,1.91) | 1.26(0.98,1.58) | 1.29(1.01,1.64) | 0.02(-0.07,0.10) |  | 0.25(0.15,0.39) | 0.43(0.27,0.67) | 0.37(0.23,0.58) | 0.38(0.24,0.59) | 0.02(-0.07,0.10) |
| Kuwait | 24.70(18.39,32.84) | 248.16(159.91,384.35) | 1.71(1.34,2.13) | 5.44(3.57,8.39) | 0.93(0.49,1.37) |  | 7.31(4.49,11.43) | 73.35(39.88,125.26) | 0.51(0.33,0.75) | 1.61(0.89,2.73) | 0.93(0.49,1.37) |
| Kyrgyzstan | 74.97(57.88,95.56) | 547.43(382.35,761.98) | 1.83(1.45,2.29) | 8.28(5.84,11.52) | 1.29(0.69,1.90) |  | 22.21(13.87,33.78) | 161.68(95.69,257.43) | 0.54(0.35,0.81) | 2.45(1.45,3.89) | 1.29(0.69,1.90) |
| Lao People's Democratic Republic | 64.60(50.02,83.93) | 157.91(118.57,207.20) | 1.78(1.41,2.24) | 2.26(1.72,2.94) | 0.14(0.02,0.26) |  | 19.15(11.70,29.50) | 46.76(29.19,69.91) | 0.53(0.33,0.80) | 0.67(0.43,0.99) | 0.14(0.02,0.26) |
| Latvia | 47.65(37.04,59.97) | 127.22(78.91,214.83) | 1.68(1.31,2.14) | 6.02(3.67,10.12) | 0.78(0.30,1.26) |  | 14.11(8.94,21.18) | 37.83(19.39,74.63) | 0.50(0.31,0.76) | 1.79(0.92,3.51) | 0.78(0.30,1.27) |
| Lebanon | 44.01(33.73,56.23) | 534.03(363.37,723.18) | 1.60(1.24,2.02) | 9.44(6.44,12.79) | 1.23(0.57,1.90) |  | 13.03(8.17,19.99) | 157.41(89.76,258.59) | 0.47(0.30,0.73) | 2.78(1.59,4.55) | 1.23(0.57,1.89) |
| Lesotho | 24.98(19.35,33.08) | 150.74(80.66,232.78) | 1.80(1.40,2.27) | 8.37(4.50,12.86) | 1.01(0.41,1.62) |  | 7.40(4.55,11.16) | 44.47(22.79,76.91) | 0.53(0.34,0.81) | 2.47(1.26,4.26) | 1.01(0.41,1.62) |
| Liberia | 39.61(30.50,52.69) | 310.69(214.13,416.59) | 1.85(1.43,2.32) | 6.24(4.33,8.27) | 1.11(0.56,1.67) |  | 11.74(7.15,17.80) | 91.99(54.74,138.43) | 0.55(0.35,0.83) | 1.85(1.10,2.81) | 1.11(0.56,1.67) |
| Libya | 43.24(34.79,55.19) | 643.02(427.18,839.77) | 1.26(1.03,1.54) | 9.44(6.29,12.27) | 2.05(1.36,2.75) |  | 12.80(8.18,19.15) | 189.94(103.29,284.70) | 0.37(0.24,0.54) | 2.78(1.52,4.17) | 2.05(1.36,2.74) |
| Lithuania | 64.56(50.02,81.37) | 236.96(147.75,352.65) | 1.68(1.31,2.15) | 7.70(4.80,11.47) | 0.95(0.38,1.52) |  | 19.12(11.99,28.66) | 70.08(37.65,116.42) | 0.50(0.31,0.76) | 2.28(1.23,3.81) | 0.95(0.38,1.52) |
| Luxembourg | 7.73(5.88,9.79) | 28.61(23.42,34.44) | 1.76(1.37,2.20) | 3.98(3.24,4.81) | 0.59(0.21,0.98) |  | 2.29(1.43,3.46) | 8.46(5.53,12.36) | 0.52(0.33,0.79) | 1.18(0.76,1.71) | 0.59(0.21,0.97) |
| Madagascar | 162.24(124.11,212.87) | 2319.92(1702.94,3011.37) | 1.52(1.19,1.90) | 8.80(6.58,11.36) | 1.44(0.71,2.18) |  | 48.09(29.38,75.60) | 686.84(417.76,1049.16) | 0.45(0.29,0.68) | 2.60(1.59,3.97) | 1.44(0.71,2.18) |
| Malawi | 131.89(100.88,173.50) | 1631.49(1131.44,2057.13) | 1.52(1.18,1.89) | 9.10(6.32,11.35) | 1.20(0.49,1.91) |  | 39.10(23.85,61.51) | 482.81(279.81,724.31) | 0.45(0.29,0.68) | 2.69(1.57,4.04) | 1.19(0.48,1.91) |
| Malaysia | 282.47(219.90,367.90) | 1128.86(876.57,1424.78) | 1.78(1.41,2.24) | 3.55(2.74,4.45) | 0.41(0.14,0.67) |  | 83.70(51.42,129.82) | 333.87(206.33,488.12) | 0.53(0.33,0.80) | 1.05(0.65,1.52) | 0.40(0.14,0.67) |
| Maldives | 3.36(2.57,4.42) | 23.27(17.59,35.17) | 1.79(1.42,2.26) | 4.56(3.49,6.95) | 0.65(0.30,1.00) |  | 0.99(0.60,1.54) | 6.88(4.26,11.73) | 0.53(0.34,0.80) | 1.35(0.84,2.29) | 0.65(0.29,1.00) |
| Mali | 139.00(107.21,184.15) | 1794.34(1154.70,2336.24) | 1.84(1.43,2.31) | 8.51(5.52,11.03) | 1.30(0.65,1.95) |  | 41.19(25.08,61.98) | 531.60(290.55,797.58) | 0.54(0.35,0.83) | 2.52(1.38,3.77) | 1.30(0.65,1.95) |
| Malta | 6.96(5.36,8.74) | 17.72(14.75,21.38) | 1.76(1.37,2.21) | 3.37(2.80,4.02) | 0.42(0.09,0.76) |  | 2.06(1.31,3.15) | 5.25(3.42,7.69) | 0.52(0.33,0.79) | 1.00(0.65,1.44) | 0.42(0.09,0.76) |
| Marshall Islands | 0.50(0.38,0.70) | 0.71(0.53,0.94) | 1.26(0.98,1.59) | 1.37(1.05,1.74) | 0.05(-0.03,0.14) |  | 0.15(0.09,0.24) | 0.21(0.13,0.32) | 0.37(0.23,0.58) | 0.41(0.25,0.61) | 0.05(-0.03,0.14) |
| Mauritania | 33.15(25.63,44.06) | 300.97(193.48,448.99) | 1.84(1.43,2.31) | 7.49(4.84,11.20) | 1.11(0.53,1.69) |  | 9.82(6.00,14.89) | 89.29(48.67,154.65) | 0.54(0.35,0.83) | 2.22(1.22,3.85) | 1.11(0.53,1.69) |
| Mauritius | 18.29(14.20,23.65) | 30.15(23.90,37.36) | 1.78(1.41,2.24) | 2.18(1.76,2.72) | 0.13(0.02,0.24) |  | 5.42(3.32,8.32) | 8.92(5.68,12.87) | 0.53(0.33,0.80) | 0.65(0.41,0.94) | 0.13(0.02,0.24) |
| Mexico | 3655.14(2926.88,4625.56) | 13678.92(11451.73,16050.70) | 4.73(3.87,5.76) | 10.60(8.88,12.44) | 0.67(0.31,1.04) |  | 1082.74(662.63,1615.33) | 4048.99(2614.57,5855.43) | 1.40(0.91,2.04) | 3.14(2.03,4.54) | 0.67(0.30,1.04) |
| Mongolia | 34.56(26.57,45.07) | 152.24(114.19,188.35) | 1.84(1.45,2.30) | 4.74(3.56,5.83) | 0.55(0.18,0.91) |  | 10.24(6.36,15.55) | 45.14(28.52,66.94) | 0.54(0.35,0.81) | 1.41(0.88,2.07) | 0.55(0.18,0.91) |
| Montenegro | 9.21(7.16,11.63) | 69.72(55.40,83.71) | 1.47(1.15,1.86) | 10.49(8.29,12.64) | 1.27(0.44,2.10) |  | 2.73(1.74,4.16) | 20.53(12.70,30.36) | 0.43(0.28,0.67) | 3.09(1.96,4.56) | 1.26(0.44,2.10) |
| Morocco | 349.82(269.61,459.66) | 2920.61(1701.66,4054.36) | 1.60(1.24,2.02) | 7.84(4.57,10.91) | 1.19(0.59,1.80) |  | 103.61(64.33,161.74) | 868.32(392.48,1467.95) | 0.47(0.30,0.73) | 2.33(1.05,3.93) | 1.19(0.59,1.80) |
| Mozambique | 182.92(140.87,239.02) | 2876.77(1994.01,3637.02) | 1.51(1.18,1.89) | 10.27(7.17,12.92) | 1.25(0.49,2.00) |  | 54.22(33.37,84.29) | 848.62(503.77,1277.79) | 0.45(0.28,0.68) | 3.01(1.76,4.59) | 1.24(0.49,1.99) |
| Myanmar | 649.30(506.83,840.69) | 2199.11(1553.96,2802.62) | 1.78(1.41,2.24) | 3.92(2.79,4.97) | 0.50(0.20,0.80) |  | 192.41(117.90,296.50) | 650.55(387.15,1002.56) | 0.53(0.33,0.80) | 1.16(0.69,1.78) | 0.50(0.20,0.80) |
| Namibia | 22.52(17.33,30.03) | 168.13(108.83,232.97) | 1.83(1.42,2.29) | 7.21(4.74,9.92) | 0.88(0.33,1.43) |  | 6.67(4.10,10.14) | 49.89(26.83,80.32) | 0.54(0.34,0.83) | 2.14(1.17,3.42) | 0.88(0.33,1.43) |
| Nepal | 375.20(295.36,484.27) | 2599.90(1968.29,3617.96) | 2.24(1.80,2.80) | 8.52(6.47,11.81) | 0.52(-0.04,1.09) |  | 111.19(68.94,167.46) | 770.82(454.50,1213.54) | 0.66(0.42,0.98) | 2.52(1.50,3.95) | 0.52(-0.05,1.09) |
| Netherlands | 229.94(177.05,294.93) | 797.53(643.98,1042.35) | 1.40(1.08,1.78) | 3.98(3.18,5.22) | 1.46(1.04,1.88) |  | 67.99(43.31,102.16) | 235.96(148.78,360.38) | 0.41(0.27,0.62) | 1.18(0.73,1.79) | 1.46(1.04,1.88) |
| New Zealand | 81.05(66.37,96.94) | 128.58(107.69,151.80) | 2.27(1.87,2.73) | 2.12(1.79,2.52) | -0.16(-0.36,0.04) |  | 23.98(15.46,34.26) | 38.04(24.99,53.98) | 0.67(0.43,0.96) | 0.63(0.41,0.88) | -0.16(-0.36,0.04) |
| Nicaragua | 140.35(106.63,187.04) | 548.19(437.82,662.19) | 3.87(3.14,4.79) | 8.52(6.82,10.28) | 0.63(0.28,0.98) |  | 41.59(24.95,63.56) | 162.31(103.81,236.84) | 1.15(0.74,1.67) | 2.52(1.61,3.67) | 0.63(0.28,0.98) |
| Niger | 125.20(96.48,169.07) | 1358.77(873.89,1940.95) | 1.84(1.43,2.32) | 6.33(4.14,8.99) | 1.02(0.49,1.55) |  | 37.11(22.58,56.96) | 402.61(233.59,650.55) | 0.55(0.35,0.83) | 1.88(1.09,3.02) | 1.02(0.49,1.55) |
| Nigeria | 1633.92(1273.74,2142.42) | 17590.88(14085.83,21150.89) | 2.04(1.61,2.57) | 8.49(6.93,10.19) | 1.24(0.62,1.86) |  | 484.37(296.02,747.92) | 5206.29(3313.99,7759.36) | 0.61(0.38,0.93) | 2.51(1.60,3.70) | 1.24(0.61,1.86) |
| North Macedonia | 28.09(21.84,35.79) | 270.39(194.07,341.10) | 1.42(1.11,1.80) | 11.47(8.21,14.48) | 1.56(0.75,2.38) |  | 8.32(5.25,12.43) | 79.43(45.43,121.97) | 0.42(0.27,0.63) | 3.37(1.99,5.11) | 1.56(0.75,2.37) |
| Northern Mariana Islands | 0.50(0.37,0.68) | 0.92(0.73,1.13) | 1.27(0.99,1.60) | 1.89(1.53,2.32) | 0.32(0.14,0.49) |  | 0.15(0.09,0.24) | 0.27(0.18,0.40) | 0.37(0.24,0.58) | 0.56(0.36,0.84) | 0.32(0.14,0.49) |
| Norway | 136.72(109.99,170.99) | 263.66(222.22,309.71) | 2.85(2.27,3.49) | 4.15(3.56,4.91) | 0.34(-0.21,0.90) |  | 40.50(26.11,60.50) | 78.05(51.34,112.90) | 0.84(0.54,1.25) | 1.23(0.81,1.78) | 0.34(-0.21,0.90) |
| Oman | 25.97(19.91,35.24) | 233.64(163.83,345.23) | 1.61(1.25,2.05) | 5.25(3.66,7.65) | 0.93(0.48,1.39) |  | 7.69(4.72,12.14) | 68.92(41.11,108.70) | 0.48(0.31,0.73) | 1.55(0.92,2.42) | 0.93(0.47,1.39) |
| Pakistan | 2418.11(1883.27,3107.34) | 18334.58(13697.39,23317.05) | 2.47(1.97,3.08) | 8.23(6.17,10.39) | 0.99(0.52,1.46) |  | 716.59(440.86,1087.11) | 5425.61(3359.49,8208.06) | 0.73(0.47,1.11) | 2.44(1.50,3.66) | 0.99(0.52,1.46) |
| Palestine | 26.42(20.08,35.28) | 487.77(313.02,642.95) | 1.60(1.24,2.02) | 10.33(6.66,13.70) | 1.29(0.59,1.99) |  | 7.83(4.81,12.04) | 143.95(78.18,227.29) | 0.47(0.30,0.73) | 3.04(1.62,4.79) | 1.28(0.59,1.98) |
| Panama | 86.33(68.62,109.63) | 330.57(264.96,401.85) | 3.92(3.17,4.84) | 7.67(6.14,9.35) | 0.65(0.33,0.97) |  | 25.58(15.94,37.81) | 97.88(63.66,143.54) | 1.16(0.75,1.69) | 2.27(1.48,3.33) | 0.65(0.33,0.97) |
| Papua New Guinea | 45.94(34.45,61.60) | 354.15(226.59,522.14) | 1.26(0.98,1.59) | 3.52(2.29,5.18) | 0.60(0.22,0.99) |  | 13.61(8.35,21.23) | 104.79(57.32,177.54) | 0.37(0.23,0.58) | 1.04(0.58,1.74) | 0.60(0.22,0.99) |
| Paraguay | 40.69(31.20,52.51) | 498.73(345.22,670.76) | 1.28(0.98,1.65) | 7.05(4.88,9.44) | 1.15(0.49,1.81) |  | 12.04(7.59,18.46) | 147.83(86.67,233.83) | 0.38(0.24,0.57) | 2.09(1.24,3.30) | 1.15(0.49,1.82) |
| Peru | 482.04(383.42,602.65) | 3035.26(2299.01,3942.48) | 2.69(2.15,3.29) | 8.39(6.38,10.91) | 1.00(0.47,1.54) |  | 142.78(90.28,211.66) | 897.88(544.73,1368.01) | 0.80(0.52,1.18) | 2.48(1.51,3.77) | 1.00(0.47,1.54) |
| Philippines | 1107.78(839.74,1470.47) | 6357.39(5207.22,7505.36) | 1.99(1.54,2.49) | 5.68(4.67,6.69) | 1.08(0.71,1.45) |  | 328.26(204.51,508.78) | 1881.93(1199.51,2759.95) | 0.59(0.37,0.89) | 1.68(1.08,2.47) | 1.08(0.71,1.45) |
| Plurinational State of Bolivia | 138.22(110.09,174.75) | 1309.20(961.25,1644.11) | 2.68(2.13,3.27) | 11.34(8.39,14.17) | 1.22(0.58,1.86) |  | 40.95(25.83,60.85) | 386.67(238.17,573.35) | 0.79(0.52,1.18) | 3.35(2.08,4.94) | 1.22(0.58,1.86) |
| Poland | 622.84(464.84,813.35) | 2794.33(2240.28,3372.12) | 1.59(1.18,2.07) | 6.58(5.21,7.98) | 0.68(0.05,1.32) |  | 184.49(115.42,287.65) | 827.23(523.79,1207.36) | 0.47(0.29,0.73) | 1.95(1.22,2.85) | 0.68(0.05,1.32) |
| Portugal | 200.43(153.04,254.89) | 507.44(410.44,629.12) | 1.75(1.37,2.20) | 4.07(3.27,5.12) | 0.56(0.17,0.95) |  | 59.35(37.09,90.33) | 150.27(95.53,218.91) | 0.52(0.33,0.79) | 1.20(0.76,1.73) | 0.56(0.17,0.95) |
| Principality of Monaco | 0.72(0.55,0.92) | 1.63(1.35,1.96) | 1.76(1.37,2.20) | 3.45(2.86,4.12) | 0.43(0.09,0.78) |  | 0.21(0.13,0.33) | 0.48(0.32,0.69) | 0.52(0.33,0.79) | 1.02(0.67,1.47) | 0.43(0.09,0.78) |
| Puerto Rico | 75.78(60.32,95.09) | 107.29(85.55,131.48) | 2.11(1.68,2.63) | 2.86(2.30,3.51) | -0.33(-0.72,0.06) |  | 22.44(14.47,33.25) | 31.75(19.94,45.00) | 0.62(0.40,0.93) | 0.85(0.54,1.20) | -0.33(-0.73,0.06) |
| Qatar | 6.04(4.39,8.28) | 186.61(121.44,242.43) | 1.62(1.25,2.06) | 6.66(4.39,8.59) | 1.32(0.72,1.93) |  | 1.79(1.09,2.80) | 55.20(30.03,82.60) | 0.48(0.31,0.74) | 1.97(1.07,2.96) | 1.32(0.72,1.92) |
| Republic of C?te d'Ivoire | 190.96(145.66,259.50) | 1811.51(1290.33,2321.69) | 1.85(1.43,2.32) | 7.18(5.19,9.17) | 1.19(0.60,1.79) |  | 56.60(34.68,86.66) | 536.36(316.13,823.98) | 0.55(0.35,0.84) | 2.12(1.25,3.23) | 1.19(0.60,1.79) |
| Republic of Cabo Verde | 5.84(4.55,7.68) | 42.07(25.91,56.47) | 1.82(1.42,2.29) | 7.54(4.64,10.11) | 1.19(0.61,1.77) |  | 1.73(1.06,2.59) | 12.47(6.57,20.21) | 0.54(0.34,0.82) | 2.24(1.18,3.61) | 1.19(0.61,1.77) |
| Republic of Korea | 2625.82(2053.40,3344.04) | 3143.93(2531.05,3878.84) | 5.83(4.72,7.28) | 5.97(4.84,7.42) | 0.02(-0.12,0.16) |  | 778.29(480.06,1191.71) | 931.19(603.40,1398.71) | 1.73(1.09,2.60) | 1.77(1.12,2.67) | 0.02(-0.12,0.16) |
| Republic of Moldova | 75.14(58.36,95.93) | 348.35(220.23,480.77) | 1.68(1.31,2.15) | 8.81(5.49,12.12) | 1.26(0.63,1.90) |  | 22.25(13.97,33.94) | 102.68(55.58,167.43) | 0.50(0.31,0.76) | 2.60(1.43,4.27) | 1.26(0.63,1.90) |
| Republic of Nauru | 0.11(0.09,0.15) | 0.23(0.18,0.30) | 1.27(0.99,1.60) | 2.18(1.73,2.76) | 0.37(0.15,0.59) |  | 0.03(0.02,0.05) | 0.07(0.04,0.10) | 0.38(0.24,0.58) | 0.65(0.41,0.95) | 0.37(0.15,0.59) |
| Republic of Niue | 0.03(0.02,0.04) | 0.02(0.02,0.03) | 1.26(0.98,1.59) | 1.26(0.98,1.59) | 0.00(-0.08,0.08) |  | 0.01(0.01,0.01) | 0.01(0.00,0.01) | 0.37(0.23,0.58) | 0.37(0.23,0.58) | 0.00(-0.08,0.08) |
| Republic of Palau | 0.18(0.13,0.23) | 0.24(0.18,0.30) | 1.26(0.98,1.59) | 1.27(0.99,1.60) | 0.01(-0.07,0.09) |  | 0.05(0.03,0.08) | 0.07(0.04,0.11) | 0.37(0.23,0.58) | 0.38(0.24,0.58) | 0.01(-0.07,0.09) |
| Republic of San Marino | 0.48(0.37,0.61) | 2.13(1.70,2.59) | 1.76(1.38,2.21) | 5.66(4.49,6.91) | 0.96(0.44,1.47) |  | 0.14(0.09,0.22) | 0.63(0.40,0.92) | 0.52(0.33,0.80) | 1.68(1.06,2.44) | 0.96(0.44,1.47) |
| Republic of the Gambia | 15.36(11.79,20.81) | 186.84(123.86,248.62) | 1.84(1.43,2.32) | 8.57(5.70,11.41) | 1.23(0.60,1.87) |  | 4.55(2.80,6.95) | 55.36(31.03,87.44) | 0.55(0.35,0.83) | 2.54(1.44,4.00) | 1.23(0.60,1.87) |
| Romania | 346.74(270.25,437.69) | 1458.27(1004.70,1955.01) | 1.42(1.11,1.79) | 6.92(4.71,9.27) | 1.13(0.51,1.75) |  | 102.67(64.76,153.23) | 432.10(239.79,664.56) | 0.42(0.27,0.63) | 2.05(1.12,3.16) | 1.13(0.51,1.75) |
| Russian Federation | 2897.42(2261.02,3624.78) | 14091.64(11390.14,17007.99) | 1.85(1.45,2.32) | 8.90(7.15,10.83) | 1.17(0.57,1.78) |  | 858.11(545.35,1285.16) | 4156.12(2652.10,6060.87) | 0.55(0.34,0.81) | 2.63(1.65,3.85) | 1.17(0.57,1.78) |
| Rwanda | 97.10(73.88,128.12) | 607.98(415.32,847.90) | 1.51(1.18,1.89) | 4.88(3.34,6.70) | 0.71(0.21,1.22) |  | 28.79(17.50,45.40) | 179.86(105.64,282.97) | 0.45(0.28,0.68) | 1.45(0.85,2.28) | 0.71(0.21,1.22) |
| Saint Kitts and Nevis | 0.84(0.67,1.08) | 1.63(1.27,2.02) | 2.11(1.67,2.62) | 2.63(2.10,3.21) | 0.40(0.08,0.71) |  | 0.25(0.16,0.37) | 0.48(0.31,0.70) | 0.62(0.40,0.93) | 0.78(0.50,1.13) | 0.40(0.08,0.71) |
| Saint Lucia | 2.63(2.09,3.43) | 7.16(5.10,9.43) | 2.10(1.67,2.62) | 3.78(2.73,4.91) | 0.43(0.11,0.75) |  | 0.78(0.49,1.17) | 2.12(1.21,3.32) | 0.62(0.40,0.93) | 1.12(0.64,1.73) | 0.43(0.11,0.75) |
| Saint Vincent and the Grenadines | 2.12(1.67,2.77) | 3.74(2.97,4.63) | 2.11(1.68,2.63) | 3.11(2.48,3.87) | 0.34(0.06,0.62) |  | 0.63(0.39,0.95) | 1.11(0.69,1.64) | 0.62(0.40,0.93) | 0.92(0.58,1.36) | 0.34(0.06,0.62) |
| Samoa | 1.93(1.44,2.56) | 2.56(1.98,3.33) | 1.26(0.98,1.59) | 1.28(1.00,1.62) | 0.01(-0.07,0.09) |  | 0.57(0.35,0.88) | 0.76(0.48,1.18) | 0.37(0.23,0.58) | 0.38(0.24,0.59) | 0.01(-0.07,0.09) |
| Sao Tome and Principe | 1.97(1.52,2.62) | 10.46(7.98,13.68) | 1.83(1.42,2.30) | 5.16(3.99,6.67) | 1.01(0.51,1.51) |  | 0.59(0.36,0.88) | 3.10(1.88,4.63) | 0.54(0.34,0.83) | 1.53(0.94,2.27) | 1.01(0.51,1.51) |
| Saudi Arabia | 207.63(157.94,280.11) | 1160.95(815.83,1652.74) | 1.61(1.24,2.04) | 3.24(2.31,4.62) | 0.67(0.36,0.98) |  | 61.50(37.76,96.66) | 343.78(200.30,546.58) | 0.48(0.31,0.73) | 0.96(0.55,1.52) | 0.67(0.36,0.98) |
| Senegal | 121.04(93.16,162.82) | 1372.04(1025.78,1794.52) | 1.84(1.43,2.31) | 9.37(7.00,12.16) | 1.22(0.57,1.87) |  | 35.87(21.90,54.11) | 405.89(240.59,607.40) | 0.54(0.35,0.83) | 2.77(1.66,4.17) | 1.22(0.57,1.87) |
| Serbia | 124.30(93.47,159.51) | 640.06(437.05,847.29) | 1.23(0.95,1.58) | 6.53(4.46,8.65) | 0.93(0.14,1.72) |  | 36.79(22.97,55.45) | 189.35(114.81,291.62) | 0.37(0.23,0.55) | 1.93(1.17,2.96) | 0.93(0.14,1.72) |
| Seychelles | 1.22(0.96,1.57) | 5.99(4.76,7.36) | 1.78(1.41,2.24) | 5.67(4.45,6.99) | 0.68(0.25,1.11) |  | 0.36(0.22,0.56) | 1.77(1.15,2.64) | 0.53(0.33,0.80) | 1.68(1.10,2.49) | 0.68(0.25,1.11) |
| Sierra Leone | 66.96(51.69,88.31) | 354.62(253.89,472.13) | 1.84(1.43,2.32) | 4.44(3.19,5.93) | 0.93(0.46,1.40) |  | 19.84(12.12,30.03) | 104.86(62.51,157.59) | 0.55(0.35,0.83) | 1.31(0.79,1.96) | 0.93(0.46,1.39) |
| Singapore | 180.67(142.72,231.84) | 355.22(284.87,441.54) | 5.82(4.72,7.27) | 6.09(4.97,7.55) | 0.05(-0.09,0.19) |  | 53.54(33.09,82.15) | 105.21(68.52,158.05) | 1.73(1.09,2.59) | 1.81(1.15,2.71) | 0.05(-0.09,0.19) |
| Slovakia | 76.78(59.65,97.50) | 368.81(273.86,541.87) | 1.41(1.10,1.79) | 6.18(4.53,9.08) | 0.91(0.34,1.49) |  | 22.74(14.41,34.07) | 109.07(66.05,169.75) | 0.42(0.27,0.63) | 1.83(1.10,2.85) | 0.91(0.33,1.49) |
| Slovenia | 29.21(22.66,36.89) | 127.11(83.07,205.18) | 1.41(1.10,1.79) | 5.40(3.47,8.60) | 0.87(0.34,1.40) |  | 8.65(5.48,12.98) | 37.69(20.30,68.39) | 0.42(0.26,0.63) | 1.60(0.86,2.91) | 0.87(0.34,1.40) |
| Socialist Republic of Viet Nam | 1089.78(844.20,1419.03) | 2161.18(1727.64,2687.61) | 1.78(1.41,2.23) | 2.13(1.71,2.63) | 0.10(0.00,0.21) |  | 322.98(197.36,497.69) | 639.97(410.26,945.13) | 0.53(0.33,0.80) | 0.63(0.41,0.92) | 0.10(0.00,0.21) |
| Solomon Islands | 3.77(2.81,5.10) | 8.04(6.08,10.53) | 1.27(0.99,1.59) | 1.30(1.02,1.66) | 0.02(-0.06,0.10) |  | 1.12(0.68,1.73) | 2.38(1.46,3.70) | 0.38(0.24,0.58) | 0.39(0.24,0.59) | 0.02(-0.07,0.10) |
| Somalia | 107.20(80.80,141.85) | 1324.22(978.81,1707.45) | 1.53(1.19,1.91) | 6.89(5.15,8.79) | 1.20(0.56,1.83) |  | 31.78(19.39,50.37) | 392.21(226.70,595.29) | 0.45(0.29,0.68) | 2.04(1.20,3.07) | 1.20(0.56,1.84) |
| South Africa | 682.56(522.87,896.64) | 4334.08(3470.40,5215.73) | 2.02(1.60,2.55) | 7.61(6.11,9.12) | 0.98(0.43,1.54) |  | 202.30(125.21,316.38) | 1282.79(807.53,1861.67) | 0.60(0.38,0.91) | 2.25(1.42,3.30) | 0.98(0.43,1.54) |
| South Sudan | 80.66(61.72,105.79) | 489.67(310.74,777.85) | 1.54(1.20,1.92) | 5.61(3.56,9.00) | 1.12(0.54,1.71) |  | 23.91(14.64,37.44) | 145.17(75.88,256.61) | 0.46(0.29,0.69) | 1.66(0.87,2.93) | 1.12(0.54,1.71) |
| Spain | 431.95(343.27,536.91) | 2398.89(1990.64,2882.75) | 1.04(0.83,1.31) | 4.48(3.70,5.47) | 2.65(2.25,3.06) |  | 127.73(79.55,182.89) | 710.20(464.76,1026.85) | 0.31(0.19,0.44) | 1.33(0.86,1.92) | 2.66(2.25,3.06) |
| Sri Lanka | 283.39(220.17,366.14) | 624.29(501.46,769.57) | 1.79(1.41,2.25) | 2.71(2.18,3.36) | 0.24(0.07,0.42) |  | 83.96(51.72,130.16) | 184.69(117.10,271.12) | 0.53(0.33,0.80) | 0.80(0.51,1.19) | 0.24(0.07,0.41) |
| Sudan | 264.67(203.42,350.35) | 2233.30(1117.04,3235.63) | 1.60(1.24,2.02) | 5.75(2.95,8.28) | 1.19(0.65,1.73) |  | 78.40(48.99,121.78) | 660.53(303.32,1078.56) | 0.47(0.30,0.73) | 1.70(0.80,2.77) | 1.19(0.65,1.73) |
| Suriname | 7.57(5.94,9.72) | 31.74(21.38,42.07) | 2.12(1.69,2.64) | 5.36(3.59,7.12) | 0.75(0.34,1.16) |  | 2.24(1.42,3.36) | 9.39(5.37,14.87) | 0.63(0.41,0.93) | 1.59(0.91,2.52) | 0.75(0.34,1.16) |
| Sweden | 159.54(123.91,199.90) | 572.60(479.31,683.09) | 1.61(1.26,2.02) | 4.70(3.93,5.60) | 2.54(2.21,2.87) |  | 47.25(29.59,69.60) | 169.55(110.09,245.44) | 0.48(0.30,0.72) | 1.39(0.91,2.02) | 2.54(2.21,2.87) |
| Switzerland | 139.62(106.68,176.33) | 370.54(308.65,450.93) | 1.76(1.38,2.21) | 3.55(2.93,4.32) | 0.49(0.14,0.84) |  | 41.33(25.97,62.31) | 109.74(70.16,160.01) | 0.52(0.33,0.79) | 1.05(0.67,1.53) | 0.49(0.14,0.84) |
| Syrian Arab Republic | 165.06(125.57,220.98) | 362.32(254.85,518.70) | 1.60(1.24,2.02) | 2.55(1.79,3.66) | 0.29(0.11,0.48) |  | 48.90(30.20,75.53) | 107.41(63.33,178.04) | 0.47(0.30,0.73) | 0.76(0.45,1.26) | 0.29(0.11,0.48) |
| Taiwan (Province of China) | 159.09(125.84,201.91) | 277.11(231.36,336.71) | 0.82(0.67,1.03) | 1.02(0.84,1.24) | 0.70(0.59,0.81) |  | 47.11(29.28,69.94) | 81.99(52.32,116.23) | 0.24(0.15,0.36) | 0.30(0.19,0.43) | 0.70(0.59,0.80) |
| Tajikistan | 86.22(65.99,112.34) | 703.10(484.43,912.34) | 1.83(1.45,2.30) | 7.38(5.15,9.55) | 1.05(0.52,1.58) |  | 25.55(15.72,38.83) | 208.22(119.02,323.89) | 0.54(0.35,0.81) | 2.19(1.25,3.38) | 1.05(0.52,1.58) |
| Thailand | 941.95(732.33,1222.29) | 1727.12(1357.76,2195.27) | 1.78(1.41,2.24) | 2.34(1.84,2.98) | 0.16(0.03,0.29) |  | 279.04(172.17,430.37) | 511.53(314.03,773.32) | 0.53(0.33,0.80) | 0.69(0.43,1.05) | 0.16(0.03,0.29) |
| Timor-Leste | 11.84(9.04,15.66) | 45.52(32.96,61.04) | 1.79(1.41,2.25) | 3.40(2.50,4.49) | 0.37(0.12,0.62) |  | 3.51(2.13,5.49) | 13.47(8.11,20.78) | 0.53(0.33,0.80) | 1.01(0.61,1.55) | 0.37(0.12,0.62) |
| Togo | 56.74(43.48,77.30) | 484.29(340.26,646.28) | 1.83(1.42,2.31) | 6.28(4.46,8.25) | 0.86(0.35,1.37) |  | 16.82(10.31,25.61) | 143.61(84.73,225.60) | 0.54(0.34,0.83) | 1.86(1.11,2.90) | 0.86(0.35,1.37) |
| Tokelau | 0.02(0.02,0.03) | 0.02(0.01,0.02) | 1.26(0.98,1.59) | 1.26(0.98,1.59) | 0.00(-0.08,0.08) |  | 0.01(0.00,0.01) | 0.01(0.00,0.01) | 0.37(0.23,0.58) | 0.37(0.23,0.58) | 0.00(-0.08,0.08) |
| Tonga | 1.14(0.86,1.50) | 1.28(0.98,1.65) | 1.26(0.98,1.59) | 1.26(0.98,1.59) | 0.00(-0.08,0.08) |  | 0.34(0.21,0.52) | 0.38(0.24,0.58) | 0.37(0.23,0.58) | 0.37(0.23,0.58) | 0.00(-0.08,0.08) |
| Trinidad and Tobago | 23.81(18.77,30.54) | 52.44(40.33,67.13) | 2.12(1.69,2.64) | 3.51(2.72,4.47) | 0.34(0.04,0.64) |  | 7.05(4.48,10.71) | 15.52(9.71,23.08) | 0.63(0.40,0.93) | 1.04(0.65,1.54) | 0.34(0.04,0.64) |
| Tunisia | 117.33(90.32,153.32) | 1148.10(701.30,1546.54) | 1.60(1.24,2.02) | 9.34(5.69,12.60) | 1.14(0.49,1.80) |  | 34.75(21.67,54.14) | 338.54(181.77,552.62) | 0.47(0.30,0.73) | 2.75(1.47,4.50) | 1.14(0.49,1.80) |
| Turkey | 907.65(703.70,1159.26) | 4980.60(3372.11,6985.20) | 1.79(1.40,2.23) | 5.70(3.88,8.03) | 0.96(0.52,1.39) |  | 268.83(166.65,404.92) | 1474.14(872.17,2428.03) | 0.53(0.33,0.79) | 1.69(1.00,2.78) | 0.95(0.52,1.39) |
| Turkmenistan | 59.70(45.81,77.80) | 377.23(263.42,503.42) | 1.83(1.45,2.30) | 7.47(5.28,9.92) | 1.05(0.52,1.59) |  | 17.69(10.89,26.93) | 111.80(66.07,180.76) | 0.54(0.35,0.81) | 2.21(1.33,3.56) | 1.05(0.52,1.59) |
| Tuvalu | 0.11(0.08,0.14) | 0.27(0.21,0.34) | 1.25(0.97,1.58) | 2.21(1.75,2.82) | 0.39(0.17,0.61) |  | 0.03(0.02,0.05) | 0.08(0.05,0.12) | 0.37(0.23,0.58) | 0.65(0.42,0.95) | 0.39(0.17,0.61) |
| Uganda | 231.50(176.13,305.75) | 2296.21(1696.45,3025.84) | 1.52(1.18,1.89) | 5.89(4.39,7.76) | 0.87(0.31,1.43) |  | 68.64(41.56,107.78) | 680.35(418.82,1050.00) | 0.45(0.29,0.68) | 1.74(1.08,2.64) | 0.87(0.31,1.43) |
| Ukraine | 1037.50(807.25,1291.21) | 3598.82(2194.89,5441.07) | 1.85(1.45,2.33) | 7.50(4.57,11.30) | 0.94(0.41,1.47) |  | 307.26(196.24,462.41) | 1062.83(569.80,1831.60) | 0.55(0.35,0.81) | 2.21(1.20,3.80) | 0.94(0.41,1.47) |
| United Arab Emirates | 24.93(18.35,34.49) | 579.18(361.74,848.60) | 1.61(1.25,2.05) | 5.89(3.65,8.66) | 0.97(0.48,1.46) |  | 7.38(4.47,11.75) | 171.14(92.73,273.84) | 0.48(0.31,0.74) | 1.74(0.96,2.77) | 0.97(0.48,1.46) |
| United Kingdom of Great Britain and Northern Ireland | 495.52(377.99,642.22) | 2811.13(2338.59,3289.33) | 0.74(0.57,0.95) | 3.69(3.04,4.37) | 3.41(2.93,3.89) |  | 146.64(91.98,224.88) | 832.33(545.16,1185.89) | 0.22(0.14,0.34) | 1.09(0.70,1.57) | 3.41(2.94,3.89) |
| United Republic of Tanzania | 296.10(224.92,404.72) | 4005.18(3105.57,4929.56) | 1.22(0.96,1.57) | 7.44(5.86,9.06) | 1.98(1.27,2.70) |  | 87.74(53.29,138.09) | 1185.48(740.27,1737.26) | 0.36(0.23,0.55) | 2.20(1.38,3.23) | 1.98(1.27,2.71) |
| United States of America | 10033.06(8206.19,12264.19) | 29505.28(25851.60,33751.66) | 3.62(2.95,4.37) | 7.26(6.27,8.27) | 0.76(0.56,0.96) |  | 2971.22(1922.03,4418.96) | 8732.46(5751.67,12209.12) | 1.07(0.69,1.59) | 2.15(1.41,3.05) | 0.76(0.55,0.96) |
| United States Virgin Islands | 2.16(1.70,2.72) | 3.82(2.96,5.07) | 2.10(1.67,2.62) | 3.81(2.95,5.12) | 0.53(0.21,0.85) |  | 0.64(0.41,0.95) | 1.13(0.70,1.70) | 0.62(0.40,0.92) | 1.13(0.70,1.70) | 0.53(0.21,0.85) |
| Uruguay | 112.55(89.77,141.03) | 201.96(167.43,243.18) | 3.31(2.65,4.13) | 5.34(4.42,6.37) | 0.28(0.03,0.52) |  | 33.33(21.54,49.82) | 59.80(38.23,86.98) | 0.98(0.63,1.46) | 1.58(1.01,2.30) | 0.28(0.03,0.52) |
| Uzbekistan | 340.18(261.58,441.18) | 2065.14(1080.42,2935.86) | 1.83(1.45,2.30) | 6.18(3.25,8.80) | 1.02(0.53,1.50) |  | 100.81(62.13,153.09) | 611.13(290.63,964.87) | 0.54(0.35,0.81) | 1.83(0.87,2.89) | 1.02(0.53,1.50) |
| Vanuatu | 1.69(1.27,2.27) | 3.85(2.92,5.11) | 1.26(0.99,1.59) | 1.34(1.04,1.70) | 0.03(-0.05,0.12) |  | 0.50(0.31,0.78) | 1.14(0.70,1.75) | 0.37(0.23,0.58) | 0.40(0.25,0.61) | 0.03(-0.05,0.12) |
| Yemen | 173.06(131.57,232.74) | 1265.68(710.34,2137.07) | 1.60(1.24,2.02) | 4.27(2.43,7.21) | 0.90(0.49,1.31) |  | 51.28(31.39,80.39) | 375.00(179.45,694.36) | 0.47(0.30,0.73) | 1.27(0.64,2.29) | 0.90(0.49,1.32) |
| Zambia | 106.25(80.56,140.30) | 1633.24(1177.57,2042.62) | 1.52(1.19,1.90) | 9.07(6.52,11.26) | 1.29(0.58,2.01) |  | 31.50(19.08,49.46) | 483.86(278.30,734.16) | 0.45(0.29,0.68) | 2.69(1.56,4.03) | 1.29(0.58,2.01) |
| Zimbabwe | 163.23(125.32,219.84) | 1294.19(700.82,1664.59) | 1.83(1.42,2.30) | 8.94(4.83,11.40) | 1.04(0.42,1.67) |  | 48.38(29.85,73.40) | 382.80(203.34,589.30) | 0.54(0.34,0.83) | 2.64(1.43,4.07) | 1.04(0.42,1.67) |
